# Characterization of Fluid Biomarkers Reveals Lysosome Dysfunction and Neurodegeneration in Neuronopathic MPS II Patients

**DOI:** 10.3390/ijms21155188

**Published:** 2020-07-22

**Authors:** Akhil Bhalla, Ritesh Ravi, Meng Fang, Annie Arguello, Sonnet S. Davis, Chi-Lu Chiu, Jessica R. Blumenfeld, Hoang N. Nguyen, Timothy K. Earr, Junhua Wang, Giuseppe Astarita, Yuda Zhu, Damian Fiore, Kimberly Scearce-Levie, Dolores Diaz, Heather Cahan, Matthew D. Troyer, Jeffrey M. Harris, Maria L. Escolar

**Affiliations:** 1 Denali Therapeutics Inc., South San Francisco, CA 94080, USA; ravi@dnli.com (R.R.); fang@dnli.com (M.F.); arguello@dnli.com (A.A.); davis@dnli.com (S.S.D.); chiu@dnli.com (C.-L.C.); jessie.blumenfeld@ucsf.edu (J.R.B.); nguyen@dnli.com (H.N.N.); earr@dnli.com (T.K.E.); jwang@dnli.com (J.W.); gastarita@gmail.com (G.A.); zhu@dnli.com (Y.Z.); fiore@dnli.com (D.F.); scearce-levie@dnli.com (K.S.-L.); diaz@dnli.com (D.D.); cahan@dnli.com (H.C.); troyer@dnli.com (M.D.T.); jharris@dnli.com (J.M.H.); 2 Department of Neuroscience, University of California, San Francisco, San Francisco, CA 94158, USA; 3 Department of Pediatrics, Children’s Hospital of Pittsburgh, Pittsburgh, PA 15224, USA

**Keywords:** mucopolysaccharidosis type II, Hunter syndrome, biomarkers, ETV:IDS, glycosaminoglycans (GAGs), heparan sulfate (HS), dermatan sulfate (DS), inflammation, lysosome dysfunction, neurodegeneration, neurofilament light chain (Nf-L), cerebrospinal fluid, GM3, gangliosides

## Abstract

Mucopolysaccharidosis type II is a lysosomal storage disorder caused by a deficiency of iduronate-2-sulfatase (IDS) and characterized by the accumulation of the primary storage substrate, glycosaminoglycans (GAGs). Understanding central nervous system (CNS) pathophysiology in neuronopathic MPS II (nMPS II) has been hindered by the lack of CNS biomarkers. Characterization of fluid biomarkers has been largely focused on evaluating GAGs in cerebrospinal fluid (CSF) and the periphery; however, GAG levels alone do not accurately reflect the broad cellular dysfunction in the brains of MPS II patients. We utilized a preclinical mouse model of MPS II, treated with a brain penetrant form of IDS (ETV:IDS) to establish the relationship between markers of primary storage and downstream pathway biomarkers in the brain and CSF. We extended the characterization of pathway and neurodegeneration biomarkers to nMPS II patient samples. In addition to the accumulation of CSF GAGs, nMPS II patients show elevated levels of lysosomal lipids, neurofilament light chain, and other biomarkers of neuronal damage and degeneration. Furthermore, we find that these biomarkers of downstream pathology are tightly correlated with heparan sulfate. Exploration of the responsiveness of not only CSF GAGs but also pathway and disease-relevant biomarkers during drug development will be crucial for monitoring disease progression, and the development of effective therapies for nMPS II.

## 1. Introduction

Mucopolysaccharidosis type II (MPS II), also known as Hunter syndrome, is a rare X-linked recessive lysosomal storage disease caused by a single gene defect resulting in a deficiency of iduronate-2 sulfatase (IDS) activity. Insufficiency or absence of IDS leads to the accumulation of its glycosaminoglycans (GAGs) substrates, notably heparan sulfate (HS) and dermatan sulfate (DS), and subsequent lysosome dysfunction in multiple organs and tissues, including the central nervous system [1,2]. The resulting clinical presentation of MPS II typically includes coarse facial features, hepatosplenomegaly, joint and skeletal involvement, cardiopulmonary disease, and both central and peripheral nervous system dysfunction.

Two-thirds of all MPS II cases are considered neuronopathic (nMPS II). These cases are characterized by progressive neurological impairment and debilitating central nervous system (CNS) deficits, including developmental delay, behavioral symptoms, and impaired cognition [3]. The age of symptom onset is variable, but onset typically occurs before 3 years of age in nMPS II cases [4], and later in non-neuronopathic cases. While both phenotypes display accumulation of GAGs relative to a control pediatric population, nMPS II patients have higher levels of GAGs in the cerebrospinal fluid (CSF) than non-neuronopathic patients [5,6], suggesting there is a meaningful relationship between CSF substrate accumulation and lysosome/cellular function in the brain. Interestingly, a host of secondary lysosomal storage products are uniquely elevated in brain tissue from MPS mouse models as well as nMPS II patients [1,7,8,9,10]. Of note, ganglioside and sphingolipid analysis from autopsied brain tissue show that individuals with increased lysosomal lipids in brain samples had the neuronopathic form of the disease [9]. These data from patients suggest that monitoring CSF levels of GAGs and lysosomal lipids could provide a window to measure the effects of a candidate therapeutic on the CNS dysfunction.

In addition to showing lysosomal dysfunction, MPS II mouse brains (as well MPS I and III) show widespread neuroinflammation and astrogliosis, as indicated by increased markers of gliosis [11,12,13], and increased inflammatory cytokines [12]. This is in agreement with the observation that plasma and CSF from MPS I patients showed significantly elevated inflammatory cytokines, including IL-1b, TNF-a, MCP-1 SDF-1a, IL-Ra, MIP-1b, IL-8, and VEGF [14,15,16].

While MRI imaging studies and post-mortem analysis of brain tissue show clear neurodegeneration in nMPS II [17,18,19], there are currently no reports of markers of neurodegeneration in biofluids from nMPS II patients. Neurofilament light chain (Nf-L), a structural protein of the neuronal cytoskeleton, is a reliable biomarker of axonal injury and neurodegeneration. Nf-L is elevated in the CSF and blood of patients across neurodegenerative diseases including Alzheimer’s disease, amyotrophic lateral sclerosis [20], and multiple sclerosis (MS) [21,22,23]. In addition, CSF and blood Nf-L levels have been shown to be responsive to disease-modifying treatment in relapsing forms of MS [24], spinal muscular atrophy [25], and lysosomal storage disorders such as neuronal ceroid lipofuscinosis type 2 (CLN2) [26], suggesting it is possible to monitor treatment effects on neurodegeneration in peripheral biofluids.

Understanding the pathophysiology underlying the CNS manifestation of disease with the help of fluid-based biomarkers that relate to cellular function beyond GAGs will be crucial for monitoring disease onset after newborn screening, monitoring disease progression, and ultimately, as biomarkers of effective therapy. Current therapies for nMPS II do not adequately control many aspects of the disease, including skeletal, cardiac, and pulmonary damage. Most importantly, they do not cross the blood brain barrier (BBB) and thus do not address the CNS manifestations of nMPS II disease [27,28]. There is a critical unmet medical need to treat nMPS II; thus, a need remains for the development of novel IDS therapies with effective brain penetration and activity. In order to support the development of new therapies to treat nMPS II, clinical biomarkers are needed to quantitatively monitor patients’ response to treatment. While reductions in CSF GAGs have been reported for investigational treatment of MPS II [29,30], the link between CSF GAG reduction and CNS benefit remains largely unanswered.

In this report, we utilized a well-characterized preclinical mouse model of MPS II treated with a novel brain Enzyme Transport Vehicle:Iduronate-2-sulfatase fusion protein (ETV:IDS) [31] to investigate the relationship between brain and CSF GAGs and downstream biomarkers of disease such as Nf-L. We show that in the CSF of nMPS II patients, there is not only an elevation of HS and DS, but also an increase in biomarkers of lysosome function (gangliosides). Importantly, to our knowledge, this is the first study to show that nMPS II patients, despite treatment with standard of care non-blood–brain barrier penetrant enzyme replacement therapy (ERT) (Elaprase™) or hematopoetic stem cell transplantation (HSCT), have elevated CSF and serum Nf-L levels. Taken together, these results open the possibility of monitoring Nf-L as a biomarker of treatment response for brain penetrant therapeutics such as ETV:IDS.

## 2. Results

### 2.1. High Degree of Correlation between Brain and CSF Glycosaminoglycan (GAG) as Well as Secondary Storage Substrates Such as Gangliosides (GM3)

**Figure 1:**
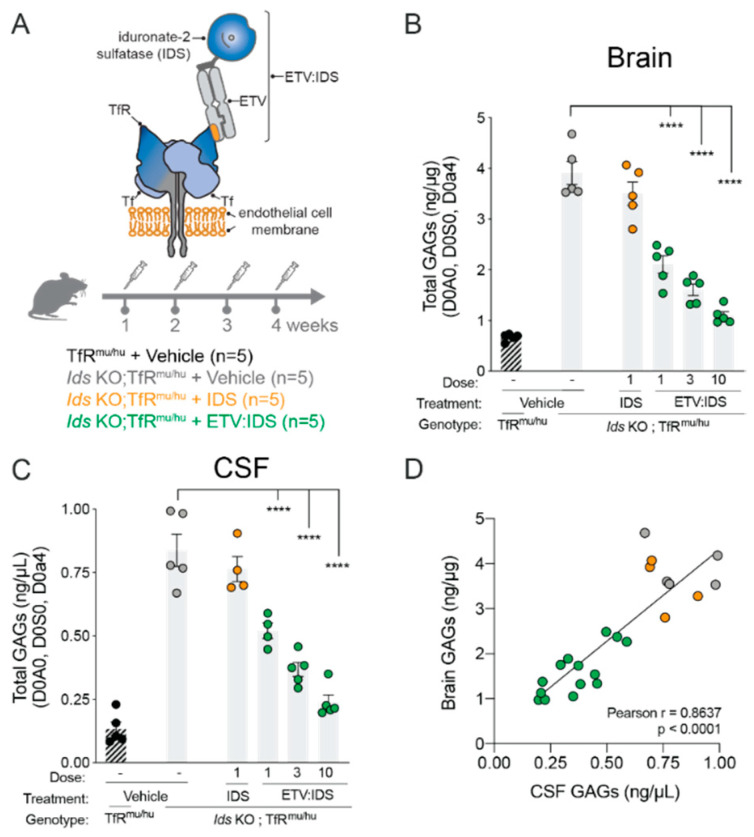
Correlation between brain and CSF GAGs in *Ids* KO;TfR^mu/hu^KI mice. GAG levels were evaluated in the brain and CSF of *Ids* KO;TfR^mu/hu^KI mice following treatment with increasing doses of ETV:IDS. TfR^mu/hu^ KI mice served as non-disease controls; (**A**) ETV:IDS is the lysosomal enzyme (E) iduronate 2-sulfatase (IDS) fused to the Transport Vehicle (TV), a TfR-binding Fc domain. *Ids* KO;TfR^mu/hu^KI were treated with 4-weekly intravenous doses of vehicle, ETV:IDS or IDS alone. Brain (**B**) and CSF (**C**) were harvested 7 days following the last dose and GAGs were measured using LC-MS/MS; (**D**) correlation between brain and CSF GAG levels of *Ids* KO;TfR^mu/hu^KI treated with ETV:IDS or IDS. Pearson r was used to determine the correlation coefficient. Graphs display mean ± SEM and *p* values: one-way ANOVA with Dunnett multiple-comparison test; **** *p* < 0.0001; *n* = 5 per group.

In order to determine the relationship between brain and CSF GAGs in the setting of IDS deficiency, we utilized a mouse model of MPS II (*Ids* KO) [32] crossed with a chimeric mouse knock-in (KI) model expressing the human TfR apical domain (*Ids* KO;TfR^mu/hu^KI; described in References [31,33]), treated with increasing doses of a TfR-mediated brain penetrant form of IDS, referred to as ETV:IDS [31]. *Ids* KO;TfR^mu/hu^KI mice received 4-weekly IV doses of vehicle, ETV:IDS or idursulfase (IDS), which does not effectively cross the blood–brain barrier (Figure 1A) [31], and GAG levels in brain and CSF were assessed seven days following the last dose using LC-MS/MS. Total GAGs were determined as the sum of the major HS (D0A0, D0S0) and DS (D0a4) derived disaccharides. TfR^mu/hu^KI mice treated with vehicle were used as controls to determine “normal” GAG levels. ETV:IDS dose-dependently lowered GAGs in both CSF and brain, while IDS alone did not reduce GAG levels. (Figure 1B,C). As previously reported [31], peripherally, IDS was equally as effective as ETV:IDS, lowering liver GAGs to control levels (data not shown). Comparison of brain and CSF GAG levels in individual *Ids* KO animals across treatment groups (vehicle, IDS and ETV:IDS) showed a significant positive correlation for ETV:IDS, suggesting that GAG levels in CSF could potentially serve as a surrogate for GAGs levels in the brain (Figure 1D).

**Figure 2:**
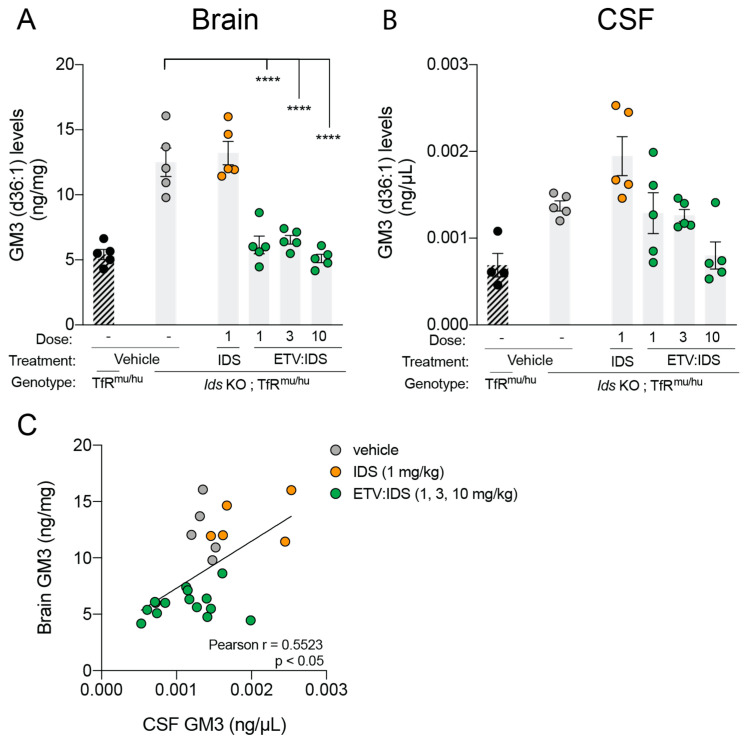
Correlation between brain and CSF gangliosides (GM3) in *Ids* KO;TfR^mu/hu^KI mice. Ganglioside GM3 levels were evaluated in brain and CSF of *Ids* KO;TfR^mu/hu^KI mice treated with IDS or increasing doses of ETV:IDS. TfR^mu/hu^KI mice served as non-disease controls. Brains (**A**) and CSF (**B**) were harvested 7 days following the last dose, and ganglioside levels were measured using LC-MS/MS; (**C**) a scatter plot was used to show the correlation between brain and CSF ganglioside levels of *Ids* KO;TfR^mu/hu^KI treated with ETV:IDS. Pearson r was used to determine the correlation coefficient. Graphs display mean ± SEM and *p* values: one-way ANOVA with Dunnett multiple-comparison test; **** *p* < 0.0001; *n* = 5 per group.

We next sought to understand the relationship between GAGs, the primary storage substrate accumulated in MPS II, and markers of secondary lysosomal dysfunction, such as gangliosides, which are ubiquitous glycosphingolipids that typically accumulate in dysfunctional lysosomes. Previous studies have shown an accumulation of lysosomal lipids in brain and CSF of *Ids* KO;TfR^mu/hu^KI and a correction of this lipid accumulation following ETV:IDS treatment [31]. Therefore, the correction of secondary lysosomal lipid storage represents strong evidence that lysosomal function is normalized. In this study, we evaluated brain and CSF ganglioside levels, in particular GM3, in *Ids* KO; TfR^mu/hu^KI mice following treatment with increasing doses of ETV:IDS or IDS at 1 mg/kg. We focused on GM3 based on previous reports which showed an increase in GM3 levels, among other lysosomal lipids, in the brains of autopsied MPS II individuals [9], as well as our recently described work in a mouse model of MPS II [31]. Similar to our previous studies, ETV:IDS significantly reduced GM3 levels in brain and CSF, while IDS alone did not (Figure 2A,B). Correlation analysis showed a significant positive correlation between brain and CSF GM3 levels, suggesting that CSF biomarker changes such as GM3 reduction could serve as a surrogate for pharmacodynamic effects in the brain, beyond primary storage products such as GAGs (Figure 2C).

**Figure 3:**
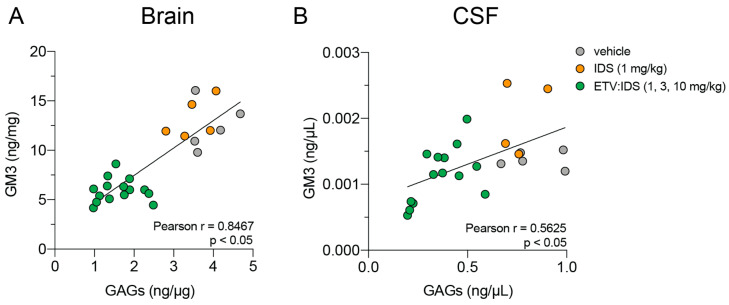
Positive correlation between primary storage substrate (GAGs) and accumulated lysosomal lipids (Ganglioside GM3) in brains and CSF *Ids* KO;TfR^mu/hu^KI mice. GAGs and Ganglioside GM3 levels were evaluated in brains (**A**) and CSF (**B**) of *Ids* KO; TfR^mu/hu^KI mice treated with increasing doses of ETV:IDS or IDS. The scatter plot was used to determine the correlation between GM3 and GAG levels in brains and CSF. Pearson r was used to determine the correlation coefficient.

We conducted additional correlation analyses to determine the relationship between primary and secondary storage biomarkers in brains and CSF. GM3 and GAG levels in brains and CSF showed a significant positive correlation (Figure 3A), albeit with a lower correlation coefficient in the CSF (Figure 3B). These data demonstrate that markers of downstream lysosomal dysfunction measured in the CSF could reflect the CNS pathophysiology of MPS II disease, thus highlighting the importance of characterizing CSF biomarkers beyond primary storage products such as GAGs.

### 2.2. nMPS II Patients on Enzyme Replacement Therapy (ERT) or Hematopoietic Stem Cell Transplant (HSCT) Show Elevated Levels of HS and DS in CSF and Serum

CSF and serum were obtained from nMPS II patients followed longitudinally at the Program for the Study of Neurodevelopment in Rare Disorders, University of Pittsburgh Medical Center, and compared to age-matched controls. The majority of patients in the nMPS II group consisted of patients who were on ERT at the time of sample collection, whereas two patients had undergone HSCT. Within the HSCT group, one of the two MPS II patients had a pre-HSCT CSF and serum sample collected at age 1 year (Table 1). CSF and serum were collected over a period of 3 years and 7 years for the HSCT patients. We were unable to make any statistical inference related to the HSCT group due to only two patients in this group. To account for repeated measures from the two HSCT patients, a linear mixed-effects model was used (see Section 4 for details).

Since HS is considered to be the primary driver of downstream pathophysiology in MPS (reviewed in Reference [34]), we evaluated HS and DS levels separately using an analytically qualified LC-MS/MS assay described in Wang et al. IJMS co-submission (Figure S1), as opposed to previously published total CSF GAG methods [29]. HS and DS levels were quantified in CSF and serum by measuring the most abundant HS derived disaccharides, D0A0 and D0S0, and DS derived disaccharide, D0a4 [35,36].

**Figure 4:**
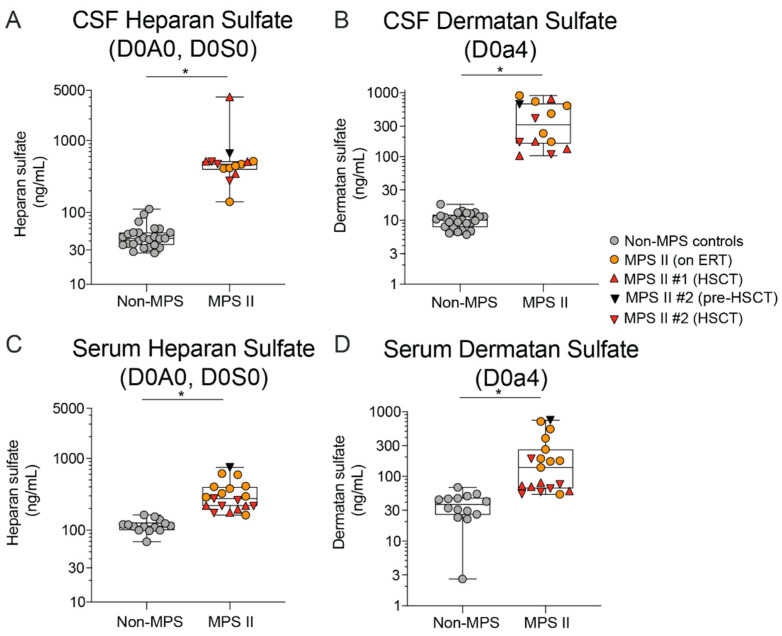
Accumulation of Heparan and Dermatan sulfate derived disaccharides in CSF and serum of MPS II patients. CSF HS (**A**) and DS (**B**) levels in MPS II patients who were either untreated at the time of sample collection (*n* = 1), on ERT (*n* = 6), or on HSCT (*n* = 2) were compared to age-matched controls (*n* = 25); serum HS **(C)** and DS **(D)** levels in MPS II patients who were either untreated at the time of sample collection (*n* = 1), on ERT (*n* = 9), or on HSCT (*n* = 2) were compared to age-matched controls. One outlier each from serum HS and serum DS (different samples) was omitted based on the Wilcoxon sum rank test [41]. Each data point represents a single control or patient visit. Data are plotted using a min to max box plot. Differences between MPS II and non-MPS controls are estimated using a linear mixed-effects model to account for repeated measures in 2 patients. No evidence of age or treatment is seen in the MPS II sample set, and thus the model will not specifically adjust for these in the estimation; * *p* < 0.05.

In agreement with the current view that standard of care ERT (Elaprase^TM^) does not cross the BBB and does not correct the CNS manifestations of nMPS II [28], we observed a significant, 11-fold and 30-fold elevation in HS and DS levels, respectively in nMPS II patient CSF relative to the control group (Figure 4A,B). In serum however, HS and DS levels were only elevated 2–3-fold over the control group, confirming that both ERT and HSCT therapies reduce peripheral GAGs in nMPS II patients (Figure 4C,D) while having little measurable effect in the CNS. In this small sample set, there did not seem to be a difference in CSF HS and DS levels between nMPS II patients on HSCT relative to patients on ERT. These data are in agreement with reports that HSCT may have less of an impact on CNS manifestations of nMPS II disease [27,37,38], in contrast to MPS I, where HSCT does seem to have a significant benefit on CNS pathology over standard ERT [39]. However, more recent studies have shown a positive impact of HSCT on CNS impairment in MPS II when treated early [40].

**Figure 5:**
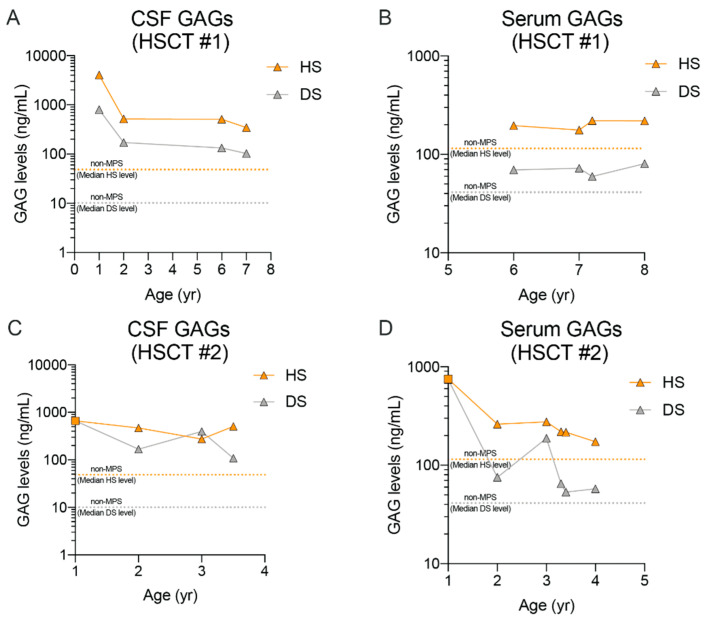
Longitudinal HS and DS levels in CSF and serum of nMPS II patients with HSCT. CSF and serum HS and DS measured by LC-MS/MS and quantified against calibration curves generated using pure reference standards for D0A0, D0S0, and D0a4. HS (orange; sum of D0A0 and D0S0) and DS (gray; D0a4) are plotted again age to show time-dependent changes in GAGs post HSCT. For HSCT patient #1 (**A**,**B**), only post-transplant samples were available, with the earliest collection at age 1. For HSCT patient # 2 (**C**,**D**), the sample collected at age 1 is a pre-HSCT sample (closed squares). Median HS and DS values for the non-MPS control group are shown for reference. Refer to Figure 4 for individual data points for control groups.

Longitudinal HS and DS analysis of the individual HSCT patients highlights interesting differences between the two patients in their HS and DS biomarker response (Figure 5). Data shows that for patient #1 there is an observed reduction in CSF HS/DS levels during the early phase of the transplant (between age 1 and 2 years) followed by a stabilization period between age 2–7 years (Figure 5A). Even at the stable period, levels of HS and DS were well above those of the control group, and in line with patients on ERT. Serum HS/DS levels in this individual are fairly constant from age 1–7 years, likely since all samples obtained were post-transplant (Figure 5B), and within 2-fold of the control group median level. In contrast, for HSCT subject #2, CSF HS/DS levels remain relatively constant from age 1–3 years even though the sample collected at age 1 is a pre-HSCT sample (Figure 5C). Serum HS and DS levels, however, were reduced following HSCT and were less than 2-fold from control levels (Figure 5D). For future analyses, it would be interesting to further characterize the relationship between neurocognitive assessment outcomes and biomarker levels in these individuals, irrespective of treatment regimen.

### 2.3. Lysosomal Lipids Are Elevated in CSF and Serum of MPS II Individuals

**Figure 6:**
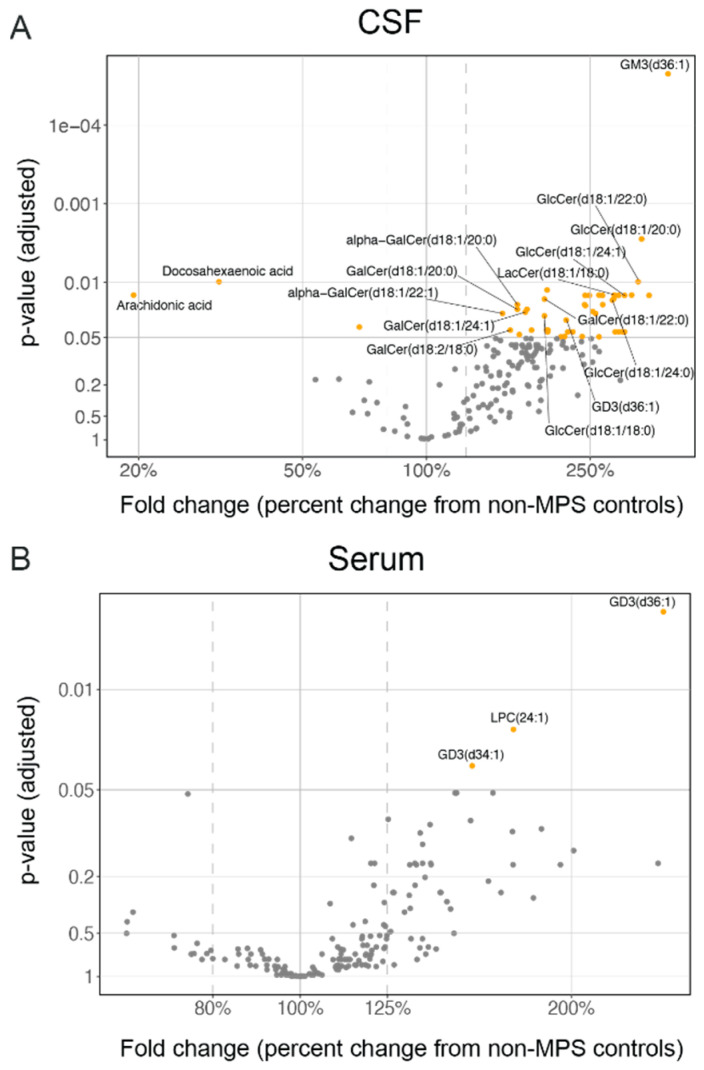
Exploratory lipidomic analysis in CSF and serum shows an accumulation of lysosomal lipids in the CSF of nMPS II patients relative to non-MPS controls. A volcano plot of lipid species in (**A**) CSF and (**B**) serum is shown. Changes in MPS II are depicted as % of non-MPS controls. Values above 100% imply an increase, while values below 100% imply a decrease from non-MPS controls. Differences between nMPS II and non-MPS controls are estimated using a linear mixed-effects model to account for subjects with repeated measures (2 MPS II patients on HSCT). Due to the exploratory nature of the analysis, age was adjusted as a linear effect across all analytes. The treatment effect was not considered due to the small number of patients with HSCT. P values are adjusted for multiple comparisons across all measurable analytes using the Benjamin-Hochberg methodology.

**Figure 7:**
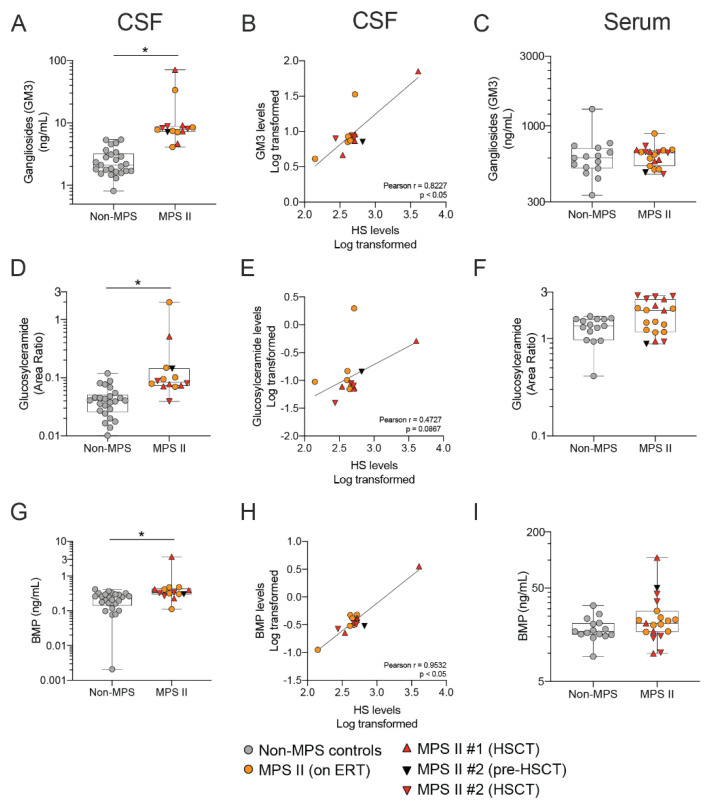
Elevated lysosomal lipid levels correlate with heparan sulfate levels in CSF of nMPS II patients. Lysosomal lipids such as Gangliosides GM3(d36:1) (**A–C**), Glucosylceramides (d18:0/20:0) (**D–F**), and BMP (18:1/18:1) (**G–I**) are quantified in CSF and serum of nMPS II patients and non-MPS controls using LC-MS/MS; (**A**,**B**,**D**,**E**,**G**,**H**) CSF lysosomal lipid levels in nMPS II patients that were either untreated at the time of sample collection (*n* = 1), on enzyme replacement therapy (*n* = 6), or on HSCT (*n* = 2) were compared to age-matched controls (*n* = 25); (**C**,**F**,**I**) serum lysosomal lipid levels in nMPS II patients that were either untreated at the time of sample collection (*n* = 1), on ERT (*n* = 9), or on HSCT (*n* = 2) were compared to age-matched controls (*n* = 15). Each data point represents a single control or patient visit. Data are plotted using a min to max box plot. Differences between nMPS II and non-MPS controls are estimated using a linear mixed-effects model to account for repeated measures in 2 patients. No evidence of age or treatment is seen in the nMPS II sample set, and thus the model will not specifically adjust for these in the estimation; * *p* < 0.05.

To identify potential translatable biomarkers of MPS II disease biology that could be used in the clinic to monitor downstream pathology, we next sought to do a broad mass-spectrometry-based lipidomic profile in nMPS II patients CSF and serum. Not surprisingly, unbiased lipid profiling showed an accumulation of numerous sphingolipid species known to be exclusively present (BMP) or enriched (GM3, GD3, and glucosylceramides) in the lysosomes (Figure 6) [42,43,44], indicative of lysosomal dysfunction in nMPS II. Lipids such as gangliosides GM3 and GD3, bis(monoacylglycerol)BMP, and glucosylceramides among others, were elevated in the CSF of nMPS II individuals relative to the control group, but this is not reflected in serum (Figure 6, Figure 7 and Figure S2). A complete list of lipids and relative changes in CSF are summarized in Table S1. Since ERT and HSCT are thought to effectively correct peripheral manifestation of nMPS pathophysiology, it is not surprising that there are far fewer significantly altered lipids in serum compared to CSF. These data are also in concordance with observations in the MPS II mouse model where *Ids* KO mice have an elevation in CSF lysosomal lipid content [31], and where a brain penetrant form of Idursulfase (ETV:IDS) is able to correct these lysosomal changes in as early as 4 weeks of dosing at clinically relevant doses. Previous studies have reported increases in ganglioside species in the brains of autopsied MPS II individuals [9], but to our knowledge, this is the first report to demonstrate alterations in fluid-based biomarkers of lysosome function that potentially reflect lysosomal dysfunction in the brain of nMPS II patients. Serum lipidomic analysis shows that the only lipid species that are altered in nMPS II patient serum are those found in lysosomes (GD3 for example), suggesting that even with ERT and HSCT, there is still some level of lysosome dysfunction present in the periphery (Figure 6B). A complete list of lipids and relative changes in serum are summarized in Table S2. Interestingly, in addition to GM3, GD3 is also seen to be elevated in the brains of MPS II patients [1,9] as well as other MPS disorders [10].

To follow up on the exploratory lipid analysis, we developed specific methods to quantify GM3, BMP, and glucosylceramides (Figure S3) since these lipids are found on lysosomes, corrected in the mouse model of MPS II, and are correlated to CSF GAGs (Figure 2,3). We also wanted to understand whether there was a correlation between HS levels in nMPS II patient CSF and levels of downstream lysosomal function. Targeted analysis of GM3, BMP, and glucosylceramides shows that nMPS II patient CSF (regardless of therapy) have elevated levels of these lipids (Figure 7A,D,G), and that there is a strong correlation between HS and lipids levels in the CSF (Figure 7B,E,H). This is in agreement with studies that show increases in heparan sulfate level may be the primary driver of downstream pathology (reviewed in [34]). Levels of these particular lysosomal lipids are not significantly different from the control group in serum, whereas other species, such as the gangliosides GD3, show a significant accumulation in nMPS II patient serum relative to the control group (Figure 6B).

### 2.4. MPS II Patient CSF and Serum Show Altered Levels of Neurodegeneration Markers Such as Nf-L

**Figure 8:**
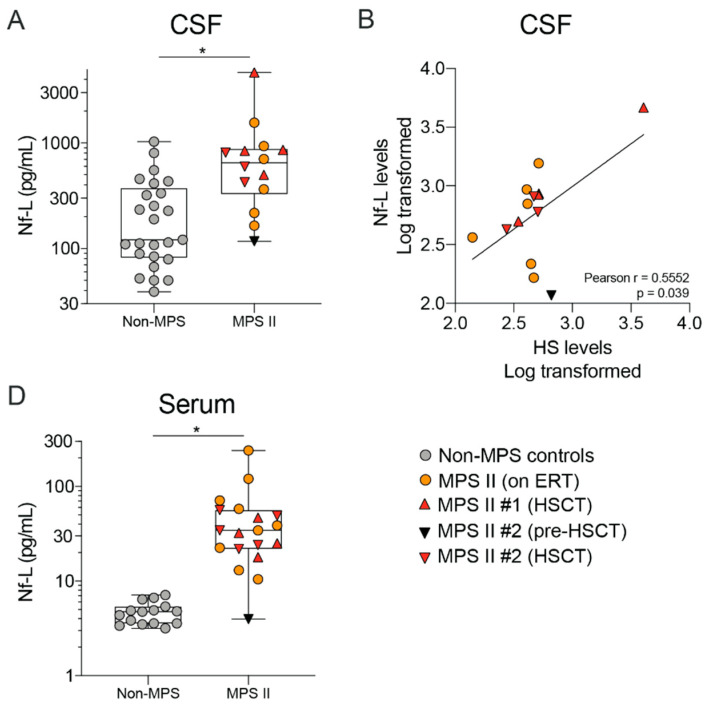
Neurofilament Light Chain (Nf-L) levels are elevated in the CSF and serum of nMPS II patients. Nf-L was quantified using SIMOA detection; (**A**,**B**) CSF Nf-L levels in nMPS II patients that were either untreated at the time of sample collection (*n* = 1), on ERT (*n* = 6), or on HSCT (*n* = 2) were compared to age-matched controls (*n* = 25); (**C**,**D**) Serum HS and DS levels in nMPS II patients who were either untreated at the time of sample collection (*n* = 1), on ERT (*n* = 9), or on HSCT (*n* = 2) were compared to age-matched controls (*n* = 15). Each data point represents a single control or patient visit. Data are plotted using a min to max box plot. Differences between nMPS II and non-MPS controls are estimated using a linear mixed-effects model to account for repeated measures in 2 patients. Age was adjusted as a linear effect, and treatment effects are not considered due to the small sample set (*n* = 2 for HSCT), and thus the model will not specifically adjust for this in the estimation; * *p* < 0.05.

We sought to determine whether we could detect alterations in a marker of neuronal degeneration neurofilament light chain (Nf-L), in this nMPS II patient population. As mentioned above, there is significant neurodegeneration in nMPS II patients, as observed via TUNEL staining of post-mortem brain tissue or MRI methods in living patients [17,18,19]. However, no fluid-based biomarker for monitoring neurodegeneration in nMPS II patients has been reported. We measured levels of Nf-L in the CSF and serum and found a significant elevation in Nf-L in both the CSF and serum of nMPS II patients regardless of their treatment regimen (Figure 8A,C), and that Nf-L levels positively correlated with CSF HS (Figure 8B). This is the first report to our knowledge that has documented an elevated neuronal injury marker in biofluids from nMPS II patients.

**Figure 9:**
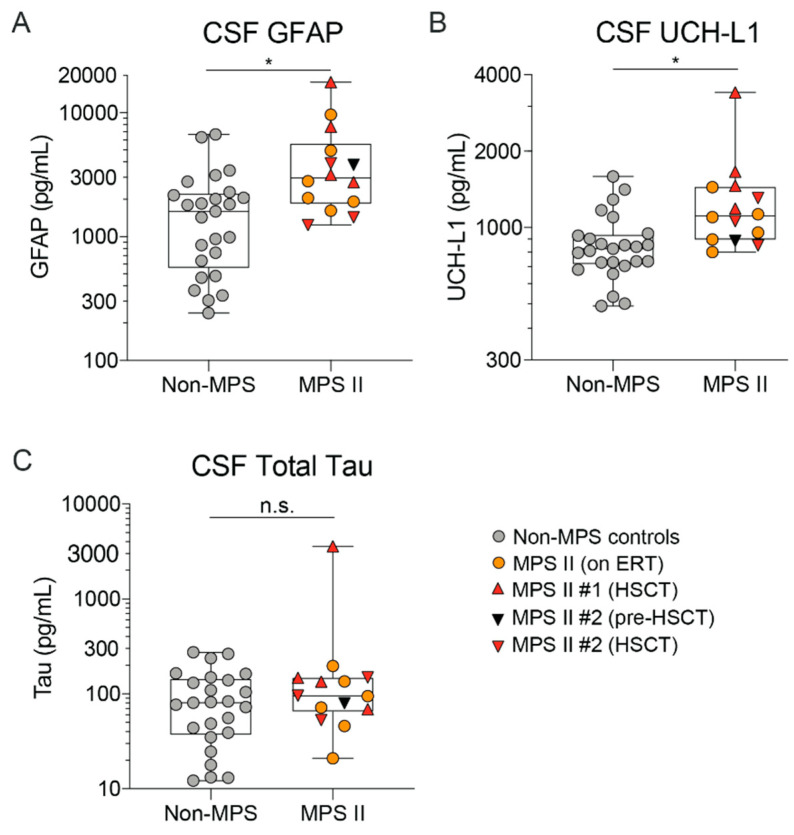
Neurodegeneration markers GFAP and UCH-L1 are elevated in nMPS II CSF. (**A**) GFAP, (**B**) UCLH-L1, and (**C**) total-Tau were quantified using SIMOA detection. nMPS II patients who were either untreated at the time of sample collection (*n* = 1), on enzyme replacement therapy (*n* = 6), or on HSCT (*n* = 2) were compared to age-matched controls (*n* = 25). Each data point represents a single control or patient visit. Data are plotted using a min to max box plot. Differences between nMPS II and non-MPS controls are estimated using a linear-mixed effects model to account for repeated measures in 2 patients. No evidence of age or treatment is seen in the MPS II sample set, and thus the model will not specifically adjust for these in the estimation; n.s.- not significant; * *p* < 0.05.

In addition to the classical fluid-based biomarkers of neurodegeneration such as Aβ_42_ and Tau, markers of neuronal health and astrogliosis such as Ubiquitin C-Terminal hydroxylase (UCH-L1) and glial fibrillary acidic protein (GFAP) are also seen to be increased in neurodegenerative disorders such as Alzheimer’s disease, ALS, and frontotemporal dementia [45,46,47,48]. Our data show that a subset of these markers is also elevated in CSF of nMPS II patients relative to the control group. We observed an increase in UCH-L1 and GFAP (Figure 9A,B), but not total Tau relative to the control group (Figure 9C). This is consistent with the absence of defined Tau pathology in MPS II [18].

## 3. Discussion

Understanding the pathophysiology of nMPS II and developing an effective treatment for neurodegeneration are pressing needs for MPS II patients and their families. Most studies have focused on either GAGs in urine and CSF (more recently HS and DS) or markers of pathway dysfunction in post-mortem brain tissue. The development of novel fluid-based biomarkers of primary storage products, downstream pathology (lysosome dysfunction and inflammation), and neurodegeneration would accelerate the field by providing a way to dynamically monitor patients’ health and response to novel therapies. The development of such novel therapeutics will be facilitated by incorporating biomarkers into the design of clinical trials to support the selection of the dose and duration of the treatment.

In this study, we utilized a well-characterized mouse model to establish the relationship between primary substrate accumulation and secondary storage biomarkers. We also showed that levels of not only GAGs, but also downstream biomarkers, are correlated between CSF and brain. Our results indicate that CSF biomarkers closely reflect storage of GAGs, accumulation of lysosomal lipids, as well as neuronal injury, and that these CSF endpoints also correlate with GAG clearance and reversal of disease markers in the *Ids* KO;TfR^mu/hu^KI mouse brain after ETV:IDS treatment.

We conducted an extensive assessment of fluid biomarkers in the CSF and serum of nMPS II patients and found that CSF, HS, and DS levels were significantly elevated regardless of ERT or HSCT, and that these levels were mostly normalized in the periphery. In addition to HS and DS, markers of lysosome function and turnover such as the lipid gangliosides, BMP, and glucosylceramides were also elevated in patient CSF. This reflects the inability of non-BBB crossing therapies to effectively correct the downstream pathophysiology of MPS II in the CNS. Our data also show the promise of fluid-based biomarkers of neuronal health in highlighting a level of neurodegeneration in nMPS II. Additional work needs to be conducted with a larger patient population in order to validate these biomarkers, as is being done for classical neurodegenerative disorders. For example, do patients with the severe neuronopathic form of MPS II have higher Nf-L, GFAP, and UCH-L1 levels than patients with the more attenuated form of disease? Future studies should try to correlate the levels of these biomarkers with clinical severity and treatment outcomes.

Since two-thirds of patients with MPS II are of the neuronopathic form, it is critical to monitor markers of neuronal health and injury in a non-invasive manner. Nf-L has emerged to be a key potential biomarker to monitor axonal injury and active neurodegeneration across multiple neurological disorders. We previously showed that Nf-L is elevated in CSF and blood in the *Ids* KO;TfR^mu/hu^KI mouse model, and normalizes after treatment with ETV:IDS [31]. In this study, we found Nf-L to be significantly elevated in both the CSF and serum of nMPS II patients on ERT and HSCT. These results suggest that monitoring long-term changes in serum Nf-L could be a non-invasive way to evaluate neuronal health and injury in nMPS II patients treated with candidate therapies to address IDS dysfunction in the CNS. In fact, Nf-L changes are currently being used to evaluate treatment response to therapy in lysosomal storage disorders such as CLN2 [26]. Future research should seek to confirm that the reduction in Nf-L can reflect a reduction in neuronal damage and degeneration in a clinical setting with therapies that cross the blood–brain barrier such as ETV:IDS. ETV:IDS achieves significant IDS exposure in the intracellular lysosomal compartment of neurons and other brain parenchymal cells [31] and is entering first-in-human trials as an investigational therapy for patients with MPS II in 2020 (https://clinicaltrials.gov/ct2/show/NCT04251026).

## 4. Materials and Methods

### 4.1. Collection of MPS II Patient and Non-MPS Control Samples

CSF and Serum from non-MPS controls were obtained from commercial sources such as Innovative Research (Novi, MI, USA), BioChemed (Winchester, VA, USA), and BioIVT (Westbury, NY, USA). MPS II patients’ CSF and serum were collected and shared by the laboratory of Dr. Maria Escolar (Children’s Hospital of Pittsburgh). Use of these samples for research was approved by the Institutional Review Board of the University of Pittsburgh (PRO11050036) on 11 January 2012. Non-MPS control group CSF and serum samples are not matched due to the limited availability in this pediatric age group. Non-MPS CSF subjects ranged from age <1 year to 16 years of age, with a median age of 5 years, whereas serum samples were obtained from individuals 1 to 9 years of age. We classified the nMPS II patient group into three distinct groups based on treatment at the time of sample collection: nMPS II patients currently on ERT (*n* = 6 for CSF; *n* = 9 for serum), nMPS II patients on no treatment (*n* = 1, with CSF and serum sample from the same patient), and nMPS II patients who underwent hematopoietic stem cell transplant around age 1 (*n* = 2). Longitudinal samples taken over the course of 4–7 years were obtained from these two patients, with the first sample being a pre-transplant (treatment naïve sample). Note that for one patient, a pre-transplant, the treatment-naïve sample was included, and that this sample is included in the no-treatment group.

*CSF collection:* CSF is drawn during a lumbar puncture. Tubes with 2 mL CSF each are taken. Tube is spun at 1970 RPM for 8 minutes, and several aliquots are made leaving about 500 µL in the tube to ensure no pelleted cells are collected. All aliquots are placed in screw top microcentrifuge tubes and frozen immediately in a −80 °C freezer and stored unless a shipment is requested. CSF samples did not go through additional freeze thaw cycles till the time of analysis.

*Serum collection:* Blood was collected via venipuncture and serum was separated after clotting as per manufacturer’s instructions. Serum samples were aliquoted and frozen immediately in a −80 °C freezer. Serum samples did not go through additional freeze-thaw cycles till the time of analysis.

### 4.2. Expression, Purification and Characterization of Recombinant Human ETV:IDS and IDS

Detailed methods for the expression and purification of ETV:IDS can be found in a previous publication [31]. Briefly, ETV:IDS were expressed as knob-in-hole bispecific proteins. ExpiCHO™ cells (Thermo Fisher Scientific, Waltham, MA USA) were transfected with plasmid DNA encoding the TV Fc polypeptide and IDS fused to an Fc polypeptide in a 1:1 plasmid ratio by weight using an Expifectamine™ CHO transfection kit according to the manufacturer’s instructions (Thermo Fisher Scientific). Idursulfase, also referred to as Elaprase™ (produced by Shire Pharmaceuticals), was purchased from WEP Clinical (Morrisville, NC, USA) and stored at 4 °C. Homogeneity of ETV:IDS and IDS (Idursulfase) were assessed by SDS-PAGE and analytical SEC-HPLC.

### 4.3. Animal Care, Generation of Mouse Strains and Dosing Methods

All procedures in animals were performed in accordance with the NIH Guide for the Care and Use of Laboratory Animals and were approved by Denali Therapeutic’s Institutional Animal Care and Use Committee on 1 March 2018. Mice were housed under a 12 h light/dark cycle and had ad libitum access to water and standard rodent diet (LabDiet^®^ #25502, Irradiated). Methods describing the generation of *Ids* KO;TfR^mu/hu^KI mice are published in References [31,32,33]. Briefly, the TfR^mu/hu^KI mouse line harboring the human TfR apical domain knocked into the mouse receptor was developed by generating a knock-in (into C57Bl/6 background) of the human apical TfR domain. *Ids* KO;TfR^mu/hu^KI mouse line was generated by breeding TfR^mu/hu^KI male mice with female *Ids* heterozygous mice to generate *Ids* KO;TfR^mu/hu^KI mice. All mice used in this study were males.

### 4.4. Preclinical Tissue and Fluid Processing for Downstream Biomarker Analysis

Detailed tissue and fluid processing methods for preclinical studies are described in Reference [31]. Briefly, brain tissue was homogenized in water and sonicated to break apart cell membranes. Tissue lysates were digested in Heparinase I, II, III, and Chondroitinase B (Iduron, Alderley Edge, UK) to digest Heparan and Dermatan sulfate (GAGs) into their disaccharides. Disaccharides were detected and quantified using the LC-MS/MS method as described in Reference [31] (Wang et al. IJMS co-submission). CSF (3 μL) was diluted in digest buffer containing enzymes as described previously. Lipid analysis was done by homogenizing 20 mg brain tissue or mixing 3 μL CSF in MS-grade methanol containing internal standards. Lipids were detected and quantified by LC-MS/MS. Detailed LC-MS/MS methods are described elsewhere [31]. MS acquisition parameters for the lipidomics assay are shown in Tables S3 and S4.

### 4.5. Nf-L and Neurology 4-Plex Analyte Analysis of Human CSF and Serum

Using Quanterix (Billerica, MA, USA) SIMOA(R) Neurofilament Light (Nf-L) Sample Diluent, cerebrospinal fluid was diluted 100× (CSF) or 4× (serum) before being added to SIMOA(R) 96-well microplate. Nf-L assay was carried out according to Quanterix SIMOA(R) Neurofilament Light Advantage Kit instructions. Sample Nf-L levels were measured using the Nf Light analysis protocol on the Quanterix SR-X instrument and interpolated against a calibration curve provided with the Quanterix assay kit. UCH-L1, GFAP, and total Tau in CSF were measured using Quanterix SIMOA(R) Neurology 4-plex kit as per manufacturer’s instructions.

### 4.6. HS and DS Analysis in Human CSF and Serum

The detailed method for the absolute quantification of HS and DS in human CSF (20 μL) and serum (5 μL) is described in (Wang et al. IJMS co-submission). Briefly, liquid chromatography (Shimadzu Nexera X2 system, Shimadzu Scientific Instrument, Columbia, MD, USA) coupled to electrospray mass spectrometry (Sciex 6500+ QTRAP, Sciex, Framingham, MA, USA) method was used for GAG measurements. For each analysis, the sample was injected on a ACQUITY UPLC BEH Amide 1.7 mm, 2.1 × 150 mm column (Waters Corporation, Milford, MA, USA) using a flow rate of 0.6 mL/min with a column temperature of 55 °C. Mobile phases A and B consisted of water with 10 mM ammonium formate and 0.1% formic acid, and acetonitrile with 0.1% formic acid, respectively. An isocratic elution was performed with 80% B throughout the 8-min run. Electrospray ionization was performed in the negative-ion mode applying the following settings: curtain gas at 20; collision gas was set at medium; ion spray voltage at −4500; temperature at 450 °C; ion source Gas 1 at 50; ion source Gas 2 at 60. Data acquisition was performed using Analyst 1.6.3 or higher (Sciex) in multiple reaction monitoring mode (MRM), with dwell time 100 ms for each species. Collision energy at −30; declustering potential at −80; entrance potential at −10; collision cell exit potential at −10. GAGs were detected as [M-H]^−^ using the following MRM transitions: D0A0 at m/z 378.1 > 87.0; D0S0 at m/z 416.1 > 138.0; D0a4 at m/z 458.1 > 300.0; D4UA-2S-GlcNCOEt-6S (HD009; Iduron, Alderley Edge, UK), at m/z 472.0 (in source fragment ion) >97.0 was used as internal standard (20 ng/sample). Individual disaccharide species were identified based on their retention times and MRM transitions using commercially-available reference standards (Iduron). GAGs were quantified via the peak area ratios of D0A0, D0S0, and D0a4 to the internal standard using Analyst 1.7.1 or MultiQuant 3.0.2 (Sciex). Reported GAG amounts were normalized to total protein levels as measured by a BCA assay (Pierce).

Pure standards for D0a4 (DS/CS), D0A0 (HS), and D0S0 (HS) were obtained from Iduron and dissolved in acetonitrile:water 50/50 (*v/v*) to generate a 1 mg/mL stock. An 8-point dilution curve in matrix (CSF or serum) was generated ranging from 5 ng/mL to 1000 ng/mL. The calibration curve with standards was treated similarly to the test samples, but without the addition of digesting enzymes.

### 4.7. LC-MS Assay for BMP, Glucosylceramides and Gangliosides

Lipids were extracted from CSF (20 μL) and serum (10 μL) by mixing in MS-grade methanol with internal standards. BMP and gangliosides analyses were performed using liquid chromatography (Shimadzu Nexera X2 system, Shimadzu Scientific Instrument, Columbia, MD, USA) coupled with electrospray mass-spectrometry (Sciex QTRAP 6500+, Sciex, Framingham, MA, USA). For each analysis, 5 µL of the sample was injected on a BEH C18 1.7 µm, 2.1 × 100 mm column (Waters Corporation, Milford, MA, USA) using a flow rate of 0.25 mL/min at 55 °C. Mobile phase A consisted of 60:40 acetonitrile/water (*v/v*) with 10 mM ammonium acetate and 0.1% acetic acid. Mobile phase B consisted of 90:10 isopropyl alcohol /acetonitrile (*v/v*) with 10 mM ammonium acetate and 0.1% acetic acid. The gradient was programmed as follows: 0.0–8.0 min from 45% B to 99% B, 8.0–9.0 min at 99% B, 9.0–9.1 min to 45% B, and 9.1–10.0 min at 45% B. Electrospray ionization was performed in negative ion mode applying the following settings: curtain gas at 30; collision gas was set at medium; ion spray voltage at −4500; temperature at 600 °C; ion source Gas 1 at 50; ion source Gas 2 at 60. Data acquisition was performed using Analyst 1.6.3 (Sciex) in multiple reaction monitoring mode (MRM) with the following parameters: dwell time (ms) for each species reported in the Tables S3 and S4, entrance potential (EP) at −10; and collision cell exit (CXP) potential at −15. BMP and gangliosides species were quantified using the non-endogenous internal standards BMP di14:0 and GM3 (d18:1/18:0(d5)). Quantification was performed using MultiQuant 3.02 (Sciex). BMP and ganglioside concentrations were normalized to either the total protein amount, tissue weight, or volume. For glucosylceramide quantification, a fraction of the methanol mixture was dried down under a stream of N2 till completely dry (>30 min), then resuspended in 92.5/5/2.5 ACN/IPA/H2O (MS grade) with 5mM ammonium formate (MS grade) and 0.5% formic acid (MS grade). Samples were sonicated in a water bath to dissolve glucoslylceramides. Glucosylceramide species were quantified using the non-endogenous internal standards GlcCer(d18:1(d5)/18:0). For each analysis, 2–5 µL of sample was injected on a HALO HILIC 2.0 µm, 3.0 × 150 mm column (Advanced Materials Technology, PN 91813-701) using a flow rate of 0.48 mL/min at 45 °C. Mobile phase A consisted of 92.5/5/2.5 ACN/IPA/H2O with 5 mM ammonium formate and 0.5% formic acid. Mobile phase B consisted of 92.5/5/2.5 H2O/IPA/ACN with 5 mM ammonium formate and 0.5% formic acid. The gradient was programmed as follows: 0.0–2 min at 100% B, 2.1 min at 95% B, 4.5 min at 85% B, hold to 6.0 min at 85% B, drop to 0% B at 6.1 min and hold to 7.0 min, ramp back to 100% at 7.1 min and hold to 8.5 min. Electrospray ionization was performed in positive ion mode applying the following settings: curtain gas at 30; collision gas was set at medium; ion spray voltage at 5500 V; temperature at 400 °C; ion source Gas 1 at 50; ion source Gas 2 at 60.

Pure standards for BMP (d18:1/18:1), BMP (di22:6), Glucosylceramide (18:1/16:0), Glucosylceramide (18:1/18:0), and GM3 (d36:1) were dissolved in methanol:IPA 50/50 (*v/v*) to generate a 1 mg/mL stock. A 8-point dilution curve in methanol:IPA 50/50 (*v/v*) was generated ranging from 0.5–100 ng/mL (GlcCer (18:1/16:0)), 1–100 ng/mL (GlcCer (18:1/18:0)), 0.1–100 ng/mL (BMP (18:1/18:1)), 1–100 ng/mL (BMP (di22:6)), 0.5–100 ng/mL (GM3 (d36:1)).

### 4.8. Exploratory Analysis of Lipids in Human CSF and Serum

CSF (20 μL) or serum (10 μL) were transferred into 1.5 mL Safe-Lock Eppendorf tubes containing a 400 µL of MS-grade methanol with internal standards. Samples were mixed vigorously using the Qiagen Tissuelyser for 30 s at 25 Hz, centrifuged for 20 min at 21,000× *g* at 4 °C and left at −20 °C for 1 h to allow for the further precipitation of proteins. Lipid analyses were performed by liquid chromatography (Shimadzu Nexera X2 system, Shimadzu Scientific Instrument, Columbia, MD, USA) coupled with electrospray mass spectrometry (QTRAP 6500+, Sciex, Framingham, MA, USA). For each analysis, 5 µL of the sample was injected on a BEH C18 1.7 µm, 2.1 × 100 mm column (Waters Corporation, Milford, MA, USA) using a flow rate of 0.25 mL/min at 55 °C. For positive ionization mode, mobile phase A consisted of 60:40 acetonitrile/water (*v/v*) with 10 mM ammonium formate and 0.1% formic acid; mobile phase B consisted of 90:10 isopropyl alcohol/acetonitrile (*v/v*) with 10 mM ammonium formate and 0.1% formic acid. For negative ionization mode, mobile phase A consisted of 60:40 acetonitrile/water (*v/v*) with 10 mM ammonium acetate and 0.1% acetic acid; mobile phase B consisted of 90:10 isopropyl alcohol/acetonitrile (*v/v*) with 10 mM ammonium acetate and 0.1% acetic acid. The gradient was programmed as follows: 0.0–8.0 min from 45% B to 99% B, 8.0–9.0 min at 99% B, 9.0–9.1 min to 45% B, and 9.1–10.0 min at 45% B. Electrospray ionization was performed in either positive or negative ion mode applying the following settings: curtain gas at 30; collision gas was set at medium; ion spray voltage at 5500 (positive mode) or −4500 (negative mode); temperature at 250 °C (positive mode) or 600 °C (negative mode); ion source Gas 1 at 55; ion source Gas 2 at 60. Data acquisition was performed using Analyst 1.6.3 (Sciex) in multiple reaction monitoring mode (MRM), with the following parameters: collision energy (CE) and declustering potential (DP) for each species reported in Table S3 (negative mode) or Table S4 (positive mode); dwell time 8 ms; EP at 10 (positive mode) or −10 (negative mode); and CXP at 12.5 (positive mode) or −15.0 (negative mode). Lipids were quantified using a mixture of non-endogenous internal standards as reported in Tables S3 and S4. Quantification was performed using MultiQuant 3.02 (Sciex).

### 4.9. Statistical Analysis of Quantified Endpoints and Exploratory Lipidomics

Results were imported into R statistical computing software. Measured values were transformed using a log base 2 transformation. Differences between MPS II and non-MPS levels in select lipids are estimated using a linear mixed-effects model [49] with the ‘nlme’ package in R [50] using subjects as random effects to account for repeated measurements. In the group comparison model for Nf-L analysis and exploratory analysis of lipids and metabolites, we adjusted for age as a continuous covariate. Due to the exploratory nature of this analysis, *p*-values are adjusted for multiple comparisons using the Benjamini-Hochberg method [51].

In both serum HS and serum DS, an outlier was observed. Sensitivity analysis was performed with and without the data point to compare the results of the two before removing outliers. When the outlier is included, the normality assumption is not met, so the Wilcoxon rank-sum test was performed [41]. Since results from both tests show a significant difference between MPS II and non-MPS population, data points are removed as outliers.

## Figures and Tables

**Table 1:** Summary of patient demographics and baseline characteristics.

	CSF			Serum		
Baseline Characteristics	Non-MPS Controls	MPS II (ERT)	MPS II (HSCT) ^a^	Non-MPS Controls	MPS II (on ERT)	MPS II (HSCT) ^a^
Gender						
Male	25 (100%)	6 (100%)	2 (100%)	7 (47%)	9 (100%)	2 (100%)
Female	0 (0%)	0 (0%)	0 (0%)	8 (53%)	0 (0%)	0 (0%)
Age (years)						
Mean (SD) ^b^	5.8 (5.4)	3.3 (1.4)	1 (0)	5.4 (2.7)	3.5 (1.2)	1 (0)
Median (min, max)	5 (<1, 16)	4 (0.5, 4)		5 (1, 9)	4 (0.5, 4)	
N (# of repeated samples)	25 (0)	6 (0)	1 (4)	15 (0)	9 (0)	1 (4)
			1 (4)			1 (6)

^a^ Two patients (siblings) underwent hematopoietic stem cell transplant (HSCT) at 9 months and 1 year of age, and longitudinal samples were collected from these patients until 3 years and 7 years of age; ^b^ for patients with repeated sampling, the mean is calculated from the age at the first sample collection.

## Supplementary Materials

Supplementary materials can be found at https://www.mdpi.com/1422-0067/21/15/5188/s1.

## Abbreviations

BBB	Blood-Brain Barrier
IDS	Iduronate 2-Sulfatase (Elaprase™)
GAG	Glycosaminoglycan
LC-MS/MS	Liquid Chromatography-Tandem Mass Spectrometry
HS	Heparan Sulfate
DS	Dermatan Sulfate
CSF	Cerebrospinal Fluid
TfR	Transferrin Receptor
MPS II	Mucopolysaccharidosis II
ETV	Enzyme Transport Vehicle
D0A0	4,5 unsaturated uronic acid residue-N-acetyl glucosamine, HS ∆UA-GluNAc
D0S0	4,5 unsaturated uronic acid residue-glucosamine-N-sulfate, ∆UA-GluNS
D0a4	4,5 unsaturated uronic acid residue-N-acetyl galactosamine, 4-O-sulfate, ∆UA-GalNAc4S
HSCT	Hematopoietic Stem Cell Transplant
ERT	Enzyme Replacement Therapy
Nf-L	Neurofilament Light Chain
TfR^mu/hu^ KI	Chimeric human/mouse Transferrin Receptor Knock-In mouse
