# Supplementary material for: Characterization of Fluid Biomarkers Reveals Lysosome Dysfunction and Neurodegeneration in Neuronopathic MPS II Patients
Source: Int J Mol Sci. 2020 Jul 22;21(15):5188. doi: 10.3390/ijms21155188 (PMC7432645; doi:10.3390/ijms21155188)
Supplement: Supplementary file 1 [file ijms-21-05188-s001.pdf]

Appendix A – Supplemental data

Table S1. CSF lipid comparison between MPS II patients and non-MPS

Table S2: Serum lipid comparison between MPS II patients and non-MPS

Table S3: MS acquisition parameters for the lipidomics assay in negative mode

Table S4: MS acquisition parameters for the lipidomics assay in positive mode

Figure S1. Standard curves for HS and DS measurements in human CSF and serum

Figure S2. Heatmap of lysosomal lipids

Figure S3. Standard curves for BMP and Gangliosides in human CSF and serum

Table S1: CSF lipid comparison between MPS II patients and non-MPS: Differences between MPS II and non-MPS II levels (shown as % of non-MPS controls) in select biomarkers are estimated using a linear mixed effects model to account for repeated measurements in 2 subjects in particular. Due to the exploratory nature of the analyses, age will be adjusted for as a linear effect across all analytes. The treatment effect is ignored here due to the small number of subjects (2) who received transplant. Due to the exploratory nature of this analysis, p-values are adjusted for multiple comparison using the Benjamini-Hochberg methodology. UL: Upper Limit of confidence interval; LL: Lower Limit of confidence interval.

Analyte	Percent of non-MPS controls	p-value (adjusted)	95% LL	95% UL
GM3(d36:1)	386	0.00002	257	581
GlcCer(d18:1/20:0)	333	0.00279	198	561
PE(P-18:0/18:2)	327	0.00977	182	586
Docosahexaenoic acid	31	0.00977	18	55
PE(P-16:0/20:4)	196	0.01244	139	277
Arachidonic acid	19	0.01445	8	46
GlcCer(d18:1/22:0)	315	0.01445	172	577
LacCer(d18:1/18:0)	286	0.01445	164	499
PC(40:5)	263	0.01445	157	439
PE(P-18:0/20:5)	348	0.01463	175	690
GlcCer(d18:1/24:1)	303	0.01463	165	558
PE(P-16:0/20:5)	293	0.01463	163	526
PI(18:0_18:1)	268	0.01463	157	458
PC(36:2)	249	0.01463	151	411
PI(18:1/18:1)	243	0.01463	151	392
PE(36:2)	285	0.01616	158	514

PE(P-18:1/20:4)	193	0.01627	133	281
GlcCer(d18:1/24:0)	283	0.01675	156	512
CE(18:2)	268	0.01911	151	476
LPC(18:0)	242	0.01911	144	406
alpha-GalCer(d18:1/20:0)	166	0.01911	124	224
PI(16:0_18:1)	243	0.01968	144	411
GalCer(d18:1/22:0)	176	0.02199	125	246
GalCer(d18:1/20:0)	166	0.02199	122	226
Cer(d18:1/24:1)	254	0.02345	144	447
GalCer(d18:1/24:1)	174	0.02376	124	244
alpha-GalCer(d18:1/22:1)	153	0.02475	118	199
LPC(20:4)	258	0.02478	144	463
GlcCer(d18:1/18:0)	194	0.02654	128	292
GD3(d36:1)	219	0.03007	132	361
PA(16:0_18:1)	69	0.03687	54	88
PE(O-18:0/20:4)	197	0.04036	125	312
PE(O-16:0/20:4)	196	0.04036	124	309
SM(d18:1/24:1)	180	0.04036	121	268
GalCer(d18:2/18:0)	160	0.04036	117	219
PE(36:4)	303	0.04240	141	652
PS(16:0_18:1)	300	0.04240	140	642
CE(20:5)	293	0.04240	139	617
PC(36:4)	288	0.04240	138	600
PC(38:4)	227	0.04240	129	398
PE(38:4)	221	0.04240	127	382
PG(18:1/18:1)	197	0.04240	123	316
PE(P-16:0/22:4)	168	0.04598	117	243
PC(O-18:0/2:0)	239	0.04859	128	445
LPC(24:1)	217	0.04859	125	376
LPC(22:6)	263	0.04911	131	527
PC(40:6)	216	0.04911	124	377
PS(16:0_20:4)	212	0.04911	124	362
PC(36:1)	175	0.05148	116	262
alpha-GalCer(d18:1/24:1)	147	0.05148	111	196
PS(18:0_20:4)	251	0.05213	128	496
SM(d18:1/24:0)	208	0.05213	121	355
Cholesterol	179	0.05213	117	276
PE(P-18:0/18:1)	151	0.05472	111	206
PS(18:0_18:1)	238	0.05640	124	456
PE(36:1)	215	0.05640	121	381
CE(20:4)	214	0.06190	119	384
PE(34:1)	200	0.06190	117	341

PI(16:0_22:6)	186	0.06190	116	300
alpha-GalCer(d18:2/22:0)	185	0.06190	115	297
PE(P-18:0/20:4)	178	0.06190	114	276
BMP(18:1/18:1)	161	0.06190	112	232
GQ1b(d36:1)	165	0.06204	112	243
PEth(18:1/18:1)	171	0.06232	112	259
alpha-GalCer(d18:1/24:0)	140	0.06381	108	183
HexCer(d18:1/24:1)	172	0.06540	112	263
Cer(d18:1/24:0)	257	0.06632	121	543
PC(O-16:0/2:0)	185	0.06632	113	303
LPC(18:1)	178	0.06632	112	282
GlcCer(d18:1/16:0)	227	0.06697	118	438
PEth(16:0_18:1)	165	0.06697	110	245
GM3(d34:1)	189	0.06808	113	315
PE(P-16:0/22:6)	175	0.06808	111	274
HexCer(d18:1/22:0)	219	0.06866	116	412
LPC(16:0)	191	0.06946	113	323
LPC(16:1)	200	0.07260	113	354
3-O-SulfoGalCer(d18:1/24:0)	146	0.07260	107	199
PC(38:6)	263	0.07543	117	588
PI(18:0_22:6)	184	0.07543	111	307
PG(18:0_18:1)	174	0.07785	109	278
PE(P-18:1/22:6)	171	0.07785	109	267
GalCer(d18:2/22:0)	198	0.07788	111	354
CE(18:1)	149	0.07862	106	209
LPI(18:0)	183	0.08141	109	307
PE(38:6)	239	0.08331	113	506
GalCer(d18:1/24:0)	181	0.08604	108	304
GalCer(d18:2/20:0)	178	0.08604	108	292
PE(P-18:0/22:6)	175	0.08614	107	286
GalCer(d18:1/22:1)	145	0.08688	105	202
PI(16:0_20:4)	176	0.09559	106	293
PI(18:0_20:4)	181	0.10198	105	312
GlcCer(d18:2/22:0)	253	0.10272	110	583
PA(18:0_22:6)	170	0.10272	104	277
SM(d18:1/16:0)	159	0.10272	104	243
PE(40:7)	241	0.11009	106	551
HexCer(d18:1/24:0)	168	0.11107	103	274
PC(34:1)	162	0.11451	102	256
3-O-SulfoGalCer(d18:1/24:1)	139	0.11911	101	192
LPE(16:0)	203	0.12046	102	402
LPE(18:0)	185	0.12046	102	337

CE(22:6)	183	0.12046	102	331
GalCer(d18:1/18:0)	133	0.12046	101	175
PE(O-18:0/22:6)	167	0.12209	101	275
PS(18:1/18:1)	215	0.12284	102	454
DG(18:0_22:6)	117	0.12670	100	137
SM(d18:1/18:0)	140	0.13422	100	196
Cer(d18:1/18:0)	150	0.13894	99	228
CE(16:1)	158	0.14875	98	255
PA(18:0_18:1)	151	0.15030	98	231
GlcCer(d18:1/22:1)	200	0.15401	98	409
GalCer(d18:1/16:0)	143	0.15401	98	210
Sphingosine	61	0.16901	36	105
LPG(18:1)	191	0.17106	94	388
Linoleic acid	54	0.17141	27	106
LacCer(d18:1/16:0)	296	0.17473	89	987
LPG(22:6)	189	0.17957	92	387
Palmitic acid	73	0.18329	51	104
LPI(16:0)	157	0.18965	94	262
alpha-GalCer(d18:1/18:0)	137	0.19296	95	196
PC(O-16:0/0:0)	108	0.19919	99	117
alpha-GalCer(d18:1/16:0)	176	0.20177	90	345
PE(40:4)	155	0.20389	92	262
3-O-SulfoGalCer(d18:1/24:0(2OH))	132	0.20560	94	185
PE(40:6)	177	0.20816	89	355
HexCer(d18:1/16:0)	188	0.21328	87	409
PE(O-16:0/22:6)	149	0.21998	91	246
Hemi-BMP(18:1/18:1)_18:0	145	0.22106	91	230
DG(18:0_20:4)	162	0.23288	88	299
alpha-GalCer(d18:2/20:0)	162	0.24597	86	305
LPC(24:0)	155	0.25823	86	278
TG(20:4_36:3)	233	0.27203	73	745
3-O-SulfoGalCer(d18:1/16:0)	130	0.27400	90	187
3-O-SulfoGalCer(d18:1/24:1(2OH))	126	0.30376	90	175
PG(16:0_18:1)	148	0.31418	83	263
DG(18:1_20:4)	190	0.31428	73	494
Oleic acid	71	0.31428	42	119
alpha-GalCer(d18:1/22:0)	123	0.33265	89	171
Palmitoleic acid	76	0.33630	49	117
PS(18:0_22:6)	177	0.34704	71	441
Hemi-BMP(22:6/22:6)_16:0	143	0.35124	80	256
Cer(d18:1/16:0)	137	0.35835	82	230
LPC(26:0)	89	0.37893	73	108

Hemi-BMP(22:6/22:6)_18:1	141	0.38644	79	254
GlcCer(d18:2/18:0)	134	0.38787	81	221
Hemi-BMP(22:6/22:6)_18:0	152	0.42415	71	325
TG(18:0_36:2)	127	0.42960	82	196
TG(20:4_34:2)	188	0.43577	58	605
DG(18:0_18:1)	66	0.44794	30	146
GlcCer(d18:2/20:0)	159	0.45342	65	392
MG(18:1)	72	0.46509	37	140
3-O-SulfoGalCer(d18:1/18:0)	117	0.46509	86	159
TG(20:4_36:2)	159	0.51242	57	442
LacCer(d18:1/24:1)	143	0.51520	64	317
GD1a/b(d36:1)	119	0.51520	81	175
Stearic acid	88	0.52715	65	118
Hemi-BMP(18:1/18:1)_16:0	127	0.53665	72	226
3-O-SulfoGalCer(d18:1/18:0(2OH)	117	0.53665	81	169
TG(22:6_36:2)	146	0.58425	54	396
PA(18:0_20:4)	90	0.62960	66	122
Hemi-BMP(18:1/18:1)_18:1	121	0.63734	69	211
BMP(20:4/20:4)	122	0.63802	68	221
PC(16:0/9:0(CHO))	132	0.71052	48	364
Hemi-BMP(22:6/22:6)_22:6	120	0.71701	60	241
TG(18:1_34:3)	132	0.72196	46	378
TG(20:4_32:1)	79	0.73263	30	204
MG(16:0)	128	0.77537	40	413
DG(16:0_20:4)	84	0.77537	36	192
PG(18:0_20:4)	120	0.78680	48	298
PE(38:5)	118	0.80408	49	286
CE HpODE	113	0.86839	45	286
TG(18:1_34:2)	111	0.89101	44	278
CE oxoHETE	90	0.90342	30	268
PC(16:0/9:0(COOH))	103	0.92217	71	148
DG(18:1/18:1)	97	0.96074	42	223
HexCer(d18:1/18:0)	99	0.96338	66	147
BMP(22:6/22:6)	101	0.96871	63	162

Table S2: Serum lipid comparison between MPS II patients and non-MPS: Differences between MPS II and non-MPS II levels (shown as % of non-MPS controls) in select biomarkers are estimated using a linear mixed effects model to account for repeated measurements in 2 subjects in particular. Due to the exploratory nature of the analyses, age is adjusted for as a linear effect across all analytes. The treatment effect is ignored here due to the small number of subjects (2) who received transplant. Due to the exploratory nature of this analysis, p-values are adjusted for multiple comparison using the Benjamini-Hochberg methodology. UL: Upper Limit of confidence interval; LL: Lower Limit of confidence interval.

Analyte	Percent of non-MPS controls	p-value (adjusted)	95% LL	95% UL
GD3(d36:1)	253	0.00292	179	356
LPC(24:1)	172	0.01928	134	222
GD3(d34:1)	155	0.03459	124	194
Sphingosine 1-phosphate	164	0.05334	124	216
HexCer(d18:1/22:0)	149	0.05334	119	187
LPC(24:0)	149	0.05334	119	186
MG(16:0)	75	0.05428	64	88
Cholesterol	125	0.08130	109	144
GlcCer(d18:2/22:0)	155	0.08318	117	204
HexCer(d18:1/24:1)	139	0.08863	112	173
LacCer(d18:1/24:0)	185	0.09495	124	278
TG(18:1_34:3)	172	0.09941	120	247
HexCer(d18:1/24:0)	136	0.10146	110	167
PC(36:2)	114	0.11057	104	125
GlcCer(d18:1/22:0)	137	0.12175	109	171
CE(20:5)	201	0.13436	121	334
BMP(22:6/22:6)	471	0.16524	158	1408
PS(18:0_18:1)	250	0.16524	124	503
PS(16:0_18:1)	194	0.16524	114	331
LacCer(d18:1/24:1)	172	0.16524	111	267
PG(16:0_18:1)	140	0.16524	106	183
GlcCer(d18:2/20:0)	140	0.16524	108	181
CE(18:2)	137	0.16524	107	174
GlcCer(d18:1/24:0)	135	0.16524	106	171
GlcCer(d18:1/22:1)	134	0.16524	107	168
3-O-SulfoGalCer(d18:1/24:0)	132	0.16524	106	166
Cer(d18:1/24:0)	121	0.16524	104	140
SM(d18:1/24:0)	120	0.16524	104	138
GlcCer(d18:1/16:0)	133	0.19452	104	169
GlcCer(d18:1/20:0)	138	0.19812	105	181
CE(22:6)	162	0.21111	106	246
Cholesterol sulfate	134	0.22173	103	175
Cer(d18:1/24:1)	121	0.22173	102	143
GM3(d36:1)	118	0.22173	102	136
GlcCer(d18:1/18:0)	135	0.22672	103	178
TG(18:1_34:2)	143	0.24575	102	200
LacCer(d18:1/16:0)	143	0.24575	102	200
HexCer(d18:1/18:0)	127	0.24575	102	159
GlcCer(d18:1/24:1)	127	0.24575	102	158
PS(18:0_20:4)	167	0.24577	103	270

PI(16:0_22:6)	132	0.25902	101	173
Glucosylsphingosine	181	0.26939	101	325
LacCer(d18:1/18:0)	145	0.28769	100	212
GM3(d34:1)	124	0.29136	100	154
LPC(26:0)	108	0.29644	100	117
LPC(26:1)	132	0.32158	99	178
PE(38:6)	147	0.32383	98	220
Arachidonic acid	65	0.34191	41	104
GlcCer(d18:2/18:0)	131	0.34191	98	175
GB3(d18:1/18:0)	118	0.39872	97	143
Coenzyme Q10	64	0.40109	38	108
CE(18:1)	124	0.42213	96	160
GB3(d18:1/16:0)	114	0.42213	97	135
PEth(18:1/18:1)	124	0.43198	95	162
PI(18:0_22:6)	126	0.47030	94	169
PC(16:0/5:0(CHO))	64	0.48460	36	113
Hemi-BMP(18:1/18:1)_18:0	148	0.48460	89	246
DG(16:0_20:4)	72	0.50330	47	111
CE(20:4)	125	0.50330	93	167
LPE(18:0)	123	0.50330	94	160
alpha-GalCer(d18:1/24:1)	120	0.50938	94	153
HexCer(d18:1/16:0)	117	0.51086	95	145
CE HpODE	137	0.53158	88	212
PE(40:6)	133	0.53158	89	198
PC(36:1)	117	0.53158	94	146
PC(34:1)	109	0.53158	97	122
DG(18:1/18:1)	125	0.53265	91	171
DG(18:1_20:4)	77	0.57339	52	113
PC(40:6)	119	0.59188	91	154
alpha-GalCer(d18:1/22:1)	117	0.59188	92	150
Cholesteryl glucoside	139	0.61101	82	234
PG(18:1/18:1)	124	0.61101	88	176
LPE(16:0)	121	0.61101	89	166
SM(d18:1/24:1)	110	0.61101	95	128
PE(P-18:0/20:4)	72	0.61833	43	122
PE(36:2)	136	0.61833	83	223
PI(16:0_20:4)	124	0.62510	87	177
LPC(20:4)	87	0.62510	70	109
LPG(18:0)	85	0.63070	65	112
PE(36:1)	141	0.63301	78	257
PEth(16:0_18:1)	111	0.63301	93	132
GB3(d18:1/24:1)	112	0.64080	92	136

GD1a/b(d36:1)	90	0.64080	76	108
PA(18:0_22:6)	80	0.64708	53	120
PE(36:4)	120	0.64708	86	167
PE(P-18:1/20:4)	76	0.68322	46	127
Docosahexaenoic acid	79	0.68322	50	124
PE(O-18:0/20:4)	76	0.68926	44	129
TG(22:6_36:2)	138	0.68997	72	262
BMP(20:4/20:4)	76	0.68997	44	132
PI(18:0_18:1)	120	0.68997	84	172
LPC(22:6)	118	0.68997	83	168
CL(72:8-2(OOH)/18:2)	85	0.68997	62	117
GB3(d18:1/24:0)	115	0.68997	86	154
alpha-GalCer(d18:2/22:0)	88	0.68997	68	114
3-O-SulfoGalCer(d18:1/24:1)	111	0.68997	91	136
LPG(18:1)	91	0.68997	75	110
SM(d18:1/16:0)	106	0.68997	94	119
3-O-SulfoGalCer(d18:1/24:0(2OH))	118	0.70214	83	169
PE(40:7)	120	0.70851	81	176
PC(40:5)	80	0.74160	48	133
CE(16:1)	119	0.74554	79	180
GalCer(d18:2/22:0)	111	0.75115	87	142
PC(38:6)	110	0.75115	88	139
3-O-SulfoGalCer(d18:1/18:0(2OH)	91	0.75115	74	113
GalCer(d18:1/18:0)	92	0.75115	76	112
Sitosteryl glucoside	135	0.75598	65	281
Eicosapentaenoic acid	78	0.75598	41	149
PE(P-16:0/20:4)	82	0.75598	50	135
alpha-GalCer(d18:2/18:0)	88	0.75598	63	123
PI(16:0_18:1)	114	0.75598	82	157
PE(38:4)	113	0.75598	83	154
Cer(d18:1/16:0)	112	0.75598	85	148
BMP(16:0_18:1)	111	0.75598	86	143
SM(d18:1/18:0)	94	0.75598	81	109
MG(16:1)	109	0.75735	87	136
PG(18:0_20:4)	116	0.77048	77	175
PS(18:0_22:6)	124	0.77943	69	222
BMP(18:1/18:1)	111	0.78359	82	150
PS(18:1/18:1)	127	0.82071	62	262
TG(18:0_36:2)	118	0.82071	71	197
Linolenic acid	117	0.82071	72	190
LPI(16:0)	115	0.82071	75	176
CE oxoHETE	115	0.82604	72	185

PE(P-18:0/22:6)	88	0.82604	58	134
Sphingosine	110	0.82604	80	151
LPC(16:0)	92	0.82604	69	121
3-O-SulfoGalCer(d18:1/24:1(2OH))	110	0.83100	80	150
MG(18:1)	119	0.83786	65	218
PE(O-16:0/20:4)	85	0.83786	48	152
Oleic acid	113	0.83786	73	176
PE(P-18:1/22:6)	89	0.83786	60	134
Hemi-BMP(18:1/18:1)_18:1	111	0.83786	76	162
TG(20:4_34:2)	111	0.83786	77	160
PG(18:0_18:1)	94	0.83786	75	117
LPG(16:0)	110	0.84083	77	158
PE(38:5)	110	0.84083	78	153
PE(P-16:0/20:5)	121	0.84442	58	253
MG(18:0)	96	0.84442	83	111
alpha-GalCer(d18:1/16:0)	95	0.84954	76	118
PC(36:4)	103	0.85347	91	118
PE(34:1)	112	0.85888	68	185
Palmitic acid	109	0.85888	74	162
PE(O-16:0/22:6)	110	0.88298	70	173
Linoleic acid	110	0.88564	71	171
GalCer(d18:2/18:0)	96	0.88564	78	118
PI(18:1/18:1)	110	0.88937	67	183
Stearic acid	92	0.88937	60	141
DG(18:0_22:6)	104	0.88937	86	124
TG(20:4_36:2)	94	0.89198	68	130
3-O-SulfoGalCer(d18:1/18:0)	96	0.91702	76	121
GalCer(d18:1/22:1)	105	0.94186	78	140
PC(O-18:0/2:0)	95	0.94726	69	131
LPC(16:1)	105	0.95210	74	149
PA(18:0_20:4)	96	0.95210	70	131
PC(O-16:0/0:0)	97	0.95210	80	118
GalCer(d18:1/20:0)	102	0.95210	85	123
PC(38:4)	98	0.95210	83	115
PE(P-16:0/22:4)	96	0.95354	71	131
PE(P-18:0/18:2)	96	0.96529	69	135
PE(P-18:0/20:5)	94	0.97077	46	193
PE(40:4)	106	0.97077	61	183
TG(20:4_32:1)	95	0.97077	53	172
LPC(18:0)	97	0.97077	70	134
TG(20:4_34:3)	103	0.97077	67	160
PA(18:1/18:1)	103	0.97077	74	145

TG(20:4_36:0)	97	0.97077	64	146
3-O-SulfoGalCer(d18:1/16:0)	97	0.97077	66	142
TG(20:4_36:3)	97	0.97077	70	134
PA(16:0_18:1)	103	0.97077	72	146
PC(O-16:0/2:0)	98	0.97077	76	126
Hemi-BMP(18:1/18:1)_16:0	98	0.97077	74	129
GalCer(d18:1/16:0)	98	0.97150	72	132
PE(O-18:0/22:6)	97	0.97194	64	147
DG(18:0_20:4)	98	0.98240	67	142
DG(16:0_18:1)	98	0.98240	70	138
PE(P-18:0/18:1)	98	0.98323	62	155
LPC(18:1)	99	0.98323	78	125
Palmitoleic acid	98	0.99381	52	186
PA(18:0_18:1)	101	0.99381	65	159
PC(16:0/9:0(CHO))	101	0.99381	64	158
PI(18:0_20:4)	99	0.99381	81	121
GalCer(d18:2/20:0)	101	0.99381	78	130
LPI(18:0)	100	0.99381	69	146
DG(18:0_18:1)	100	0.99381	72	140
Cer(d18:1/18:0)	100	0.99381	74	135
Galactosylsphingosine	100	0.99381	80	125
PE(P-16:0/22:6)	100	0.99897	67	150

Table S3: MS acquisition parameters for the lipidomics assay in negative mode: QTRAP 6500+ MS source parameters were as follows: ion spray voltage, -4500V; temperature, 600°C; curtain gas, 40 psi; collision gas, medium; ion source Gas 1, 55 psi; ion source Gas 2, 60 psi; entrance potential, -10 V; and collision cell exit potential, -15 V. Data acquisition was performed in multiple reaction monitoring mode (MRM) with the precursor ion mass-to-charge ratio (Q1 m/z); fragment ion mass-to-charge ratio (Q3 m/z); collision energy (CE) and declustering potential (DP) values are reported ~~in Table S3~~.

**Lipid**	**Internal Standard**	**Q1 m/z**	**Q3 m/z**	**DP (V)**	**CE (V)**
(3-O-sulfo)GalCer(d18:1/16:0)	(3-O-sulfo)GalCer(d18:1/18:0(d3))	778.5	97	-80	-150
(3-O-sulfo)GalCer(d18:1/18:0(2OH)	(3-O-sulfo)GalCer(d18:1/18:0(d3))	822.6	97	-80	-150
(3-O-sulfo)GalCer(d18:1/18:0(d3))	N/A	809.6	97	-80	-150
(3-O-sulfo)GalCer(d18:1/18:0)	(3-O-sulfo)GalCer(d18:1/18:0(d3))	806.6	97	-80	-150
(3-O-sulfo)GalCer(d18:1/24:0(2OH))	(3-O-sulfo)GalCer(d18:1/18:0(d3))	906.7	97	-80	-150

(3-O-sulfo)GalCer(d18:1/24:0)	(3-O-sulfo)GalCer(d18:1/18:0(d3))	890.7	97	-80	-150
(3-O-sulfo)GalCer(d18:1/24:1(2OH))	(3-O-sulfo)GalCer(d18:1/18:0(d3))	904.7	97	-80	-150
(3-O-sulfo)GalCer(d18:1/24:1)	(3-O-sulfo)GalCer(d18:1/18:0(d3))	888.7	97	-80	-150
Arachidonic acid	Arachidonic acid-d8	303.2	303.2	-80	-10
Arachidonic acid_MRM	Arachidonic acid-d8_MRM	303.2	259.1	-80	-19
Arachidonic acid-d8	N/A	311.3	311.3	-80	-10
Arachidonic acid-d8_MRM	N/A	311.3	267.1	-80	-19
BMP(14:0/14:0)	N/A	665.3	227.2	-60	-50
BMP(16:0_18:1)	BMP(14:0/14:0)	747.5	255.4	-80	-50
BMP(16:0_20:4)	BMP(14:0/14:0)	769.5	255.4	-80	-50
BMP(16:0_22:6)	BMP(14:0/14:0)	795.5	255.4	-80	-50
BMP(16:1/16:1)	BMP(14:0/14:0)	717.5	253.1	-60	-50
BMP(18:0_18:1)	BMP(14:0/14:0)	775.5	281.4	-80	-50
BMP(18:0_20:4)	BMP(14:0/14:0)	797.5	283.4	-80	-50
BMP(18:0_22:6)	BMP(14:0/14:0)	823.5	283.4	-80	-50
BMP(18:1/18:1)	BMP(14:0/14:0)	773.5	281.3	-80	-50
BMP(20:4/20:4)	BMP(14:0/14:0)	817.5	303.3	-60	-50
BMP(22:6/22:6)	BMP(14:0/14:0)	865.5	327.3	-60	-50
Cholesterol sulfate	(3-O-sulfo)GalCer(d18:1/18:0(d3))	465.3	96.7	-80	-80
CL(14:0/14:0/14:0/14:0)	N/A	619.5	227.2	-80	-50
CL(72:6/18:2)	CL(14:0/14:0/14:0/14:0)	725.7	279.2	-80	-50
CL(72:7/18:2)	CL(14:0/14:0/14:0/14:0)	724.7	279.2	-80	-50

CL(72:8-2(OOH)/18:2)	CL(14:0/14:0/14:0/14:0)	755.7	279.2	-80	-50
CL(72:8/18:2)	CL(14:0/14:0/14:0/14:0)	723.7	279.3	-80	-50
CL(74:9/18:2)	CL(14:0/14:0/14:0/14:0)	736.7	279.2	-80	-50
DHA	Arachidonic acid-d8_MRM	327.2	229.1	-80	-19
EPA	Arachidonic acid-d8_MRM	301.3	257.1	-80	-19
FAHFA(18:1/9-O-18:0)	PG(15:0/18:1(d7))	563.6	281	-60	-50
GD1a/b(d36:1)	GM3(d18:1/18:0(d5))	917.5	290.1	-60	-65
GD3(d34:1)	GM3(d18:1/18:0(d5))	720.9	290.1	-60	-65
GD3(d36:1)	GM3(d18:1/18:0(d5))	734.9	290.1	-60	-65
GM3(d18:1/18:0(d5))	N/A	1184.8	290.1	-60	-65
GM3(d34:1)	GM3(d18:1/18:0(d5))	1151.7	290.1	-60	-65
GM3(d36:1)	GM3(d18:1/18:0(d5))	1179.8	290.1	-60	-65
GQ1b(d36:1)	GM3(d18:1/18:0(d5))	1208.6	290.1	-60	-65
Hemi-BMP(14:0/14:0)_14:0	N/A	875.5	227.3	-50	-50
Hemi-BMP(18:1/18:1)_16:0	Hemi-BMP(14:0/14:0)_14:0	1011.7	281.3	-50	-50
Hemi-BMP(18:1/18:1)_18:0	Hemi-BMP(14:0/14:0)_14:0	1039.7	281.3	-50	-50
Hemi-BMP(18:1/18:1)_18:1	Hemi-BMP(14:0/14:0)_14:0	1037.7	281.3	-50	-50
Hemi-BMP(20:4/20:4)_16:0	Hemi-BMP(14:0/14:0)_14:0	1056.8	303.3	-50	-50
Hemi-BMP(20:4/20:4)_18:0	Hemi-BMP(14:0/14:0)_14:0	1185.8	303.3	-50	-50
Hemi-BMP(20:4/20:4)_18:1	Hemi-BMP(14:0/14:0)_14:0	1183.8	303.3	-50	-50
Hemi-BMP(20:4/20:4)_20:4	Hemi-BMP(14:0/14:0)_14:0	1104.8	303.3	-50	-50
Hemi-BMP(22:6/22:6)_16:0	Hemi-BMP(14:0/14:0)_14:0	1103.7	327.3	-50	-50

Hemi-BMP(22:6/22:6)_18:0	Hemi-BMP(14:0/14:0)_14:0	1131.7	327.3	-50	-50
Hemi-BMP(22:6/22:6)_18:1	Hemi-BMP(14:0/14:0)_14:0	1129.7	327.3	-50	-50
Hemi-BMP(22:6/22:6)_22:6	Hemi-BMP(14:0/14:0)_14:0	1175.7	327.3	-50	-50
Linoleic acid	Arachidonic acid-d8	279.2	279.2	-80	-38
Linolenic acid	Arachidonic acid-d8	277.2	277.2	-80	-10
LPE(16:0)	LPE(18:1(d7))	452.2	255.3	-80	-50
LPE(18:0)	LPE(18:1(d7))	480.31	283.3	-80	-50
LPE(18:1(d7))	N/A	485.3	288.3	-80	-50
LPG(16:0)	LPE(18:1(d7))	483.3	255.3	-80	-50
LPG(18:0)	LPE(18:1(d7))	511.3	283.3	-80	-50
LPG(18:1)	LPE(18:1(d7))	509.3	281.3	-80	-50
LPG(20:4)	LPE(18:1(d7))	531.3	303.3	-80	-50
LPG(22:6)	LPE(18:1(d7))	555.3	327.3	-80	-50
LPI(16:0)	LPE(18:1(d7))	571.3	241.1	-80	-50
LPI(18:0)	LPE(18:1(d7))	599.3	241.1	-80	-50
Oleic acid	Arachidonic acid-d8	281.2	281.2	-80	-10
PA(15:0/18:1(d7))	N/A	666.52	241.3	-80	-50
PA(16:0_18:1)	PA(15:0/18:1(d7))	673.5	255.3	-80	-50
PA(18:0_18:1)	PA(15:0/18:1(d7))	701.5	283.3	-80	-50
PA(18:0_20:4)	PA(15:0/18:1(d7))	723.5	283.3	-80	-50
PA(18:0_22:6)	PA(15:0/18:1(d7))	747.5	283.3	-80	-50
PA(18:1/18:1)	PA(15:0/18:1(d7))	699.5	281.3	-80	-50

Palmitic acid	Arachidonic acid-d8	255.1	255.1	-80	-10
Palmitoleic acid	Arachidonic acid-d8	253.1	253.1	-80	-10
PE(15:0/18:1(d7))	N/A	709.6	241.3	-80	-50
PE(O-16:0/20:4)	PE(15:0/18:1(d7))	724.5	303.2	-80	-50
PE(O-16:0/22:6)	PE(15:0/18:1(d7))	748.5	327.2	-80	-50
PE(O-18:0/20:4)	PE(15:0/18:1(d7))	752.6	303.2	-80	-50
PE(O-18:0/22:6)	PE(15:0/18:1(d7))	776.6	327.2	-80	-50
PE(P-16:0/20:4)	PE(15:0/18:1(d7))	722.6	303.3	-80	-50
PE(P-16:0/20:5)	PE(15:0/18:1(d7))	720.6	301.3	-80	-50
PE(P-16:0/22:4)	PE(15:0/18:1(d7))	750.6	331.3	-80	-50
PE(P-16:0/22:6)	PE(15:0/18:1(d7))	746.6	327.3	-80	-50
PE(P-18:0/18:1)	PE(15:0/18:1(d7))	728.6	281.3	-80	-50
PE(P-18:0/18:2)	PE(15:0/18:1(d7))	726.6	279.2	-80	-50
PE(P-18:0/20:4)	PE(15:0/18:1(d7))	750.6	303.3	-80	-50
PE(P-18:0/20:5)	PE(15:0/18:1(d7))	748.6	301.3	-80	-50
PE(P-18:0/22:6)	PE(15:0/18:1(d7))	774.6	327.3	-80	-50
PE(P-18:1/20:4)	PE(15:0/18:1(d7))	748.5	303.3	-80	-50
PE(P-18:1/22:6)	PE(15:0/18:1(d7))	772.5	327.3	-80	-50
PEth(16:0_18:1)	PE(15:0/18:1(d7))	772.5	255.1	-80	-50
PEth(18:1/18:1)	PE(15:0/18:1(d7))	773.6	281.2	-80	-50
PG(15:0/18:1(d7))	N/A	740.55	241.3	-80	-50
PG(16:0_18:1)	PG(15:0/18:1(d7))	747.5	255.3	-80	-50

PG(16:0_20:4)	PG(15:0/18:1(d7))	769.5	255.3	-80	-50
PG(16:0_22:6)	PG(15:0/18:1(d7))	795.5	255.3	-80	-50
PG(18:0_18:1)	PG(15:0/18:1(d7))	775.5	281.3	-80	-50
PG(18:0_20:4)	PG(15:0/18:1(d7))	797.5	283.3	-80	-50
PG(18:0_22:6)	PG(15:0/18:1(d7))	823.5	283.3	-80	-50
PG(18:1/18:1)	PG(15:0/18:1(d7))	773.4	281.4	-80	-50
PI(15:0/18:1(d7))	N/A	828.6	241.3	-80	-50
PI(16:0_18:1)	PI(15:0/18:1(d7))	835.6	255.3	-80	-50
PI(16:0_20:4)	PI(15:0/18:1(d7))	857.6	255.3	-80	-50
PI(16:0_22:6)	PI(15:0/18:1(d7))	881.6	255.3	-80	-50
PI(18:0_18:1)	PI(15:0/18:1(d7))	863.6	283.3	-80	-50
PI(18:0_20:4)	PI(15:0/18:1(d7))	885.6	283.3	-80	-50
PI(18:0_22:6)	PI(15:0/18:1(d7))	909.6	283.3	-80	-50
PI(18:1/18:1)	PI(15:0/18:1(d7))	861.6	281.3	-80	-50
PI(20:4/20:4)	PI(15:0/18:1(d7))	905.6	303.3	-80	-50
PS(15:0/18:1(d7))	N/A	753.55	241.3	-80	-50
PS(16:0_18:1)	PS(15:0/18:1(d7))	760.6	255.3	-80	-50
PS(16:0_20:4)	PS(15:0/18:1(d7))	782.6	255.3	-80	-50
PS(16:0_22:6)	PS(15:0/18:1(d7))	806.6	255.3	-80	-50
PS(18:0_18:1)	PS(15:0/18:1(d7))	788.6	283.3	-80	-50
PS(18:0_20:4)	PS(15:0/18:1(d7))	810.6	283.3	-80	-50
PS(18:0_22:6)	PS(15:0/18:1(d7))	834.6	283.3	-80	-50

PS(18:1/18:1)	PS(15:0/18:1(d7))	786.6	281.3	-80	-50
PS(22:6/22:6)	PS(15:0/18:1(d7))	878.5	327.3	-80	-50
Stearic acid	Arachidonic acid-d8	283.2	283.2	-80	-10

Table S4: MS acquisition parameters for the lipidomics assay in positive mode: QTRAP 6500+ MS source parameters were as follows: ion spray voltage, 5500V; temperature, 250°C; curtain gas, 40 psi; collision gas, medium; ion source Gas 1, 55 psi; ion source Gas 2, 60 psi; entrance potential, 10 V; and collision cell exit potential, 12.5 V. Data acquisition was performed in multiple reaction monitoring mode (MRM) with the precursor ion mass-to-charge ratio (Q1 m/z); fragment ion mass-to-charge ratio (Q3 m/z); collision energy (CE) and declustering potential (DP) values are reported ~~in Table S4~~.

Lipid	Internal Standard	Q1 m/z	Q3 m/z	DP (V)	CE (V)
1-O-Palmitoyl-Cer(d18:1/18:0)	Cer(d18:1/16:0(d7))	786.8	502.5	80	35
24-Hydroxycholesterol	24-Hydroxcholesterol-d7	385.3	367.3	80	30
24-Hydroxycholesterol(d7)	N/A	392.3	367.3	80	30
3-O-SulfoLacCer(d18:1/18:0)	LacCer(d18:1/17:0)	970.8	548.5	68	61
4-beta-Hydroxycholesterol	Cholesterol(d7)	420.3	385.3	80	15
7-keto-Cholesterol	Cholesterol(d7)	401.3	383.3	80	15
CE HETE	CE(18:1(d7))	706.6	369.2	80	25
CE HODE	CE(18:1(d7))	682.6	369.2	80	25
CE HpODE	CE(18:1(d7))	698.6	369.2	80	25
CE oxoHETE	CE(18:1(d7))	704.6	369.2	80	25
CE oxoODE	CE(18:1(d7))	680.6	369.2	80	25
CE(16:1)	CE(18:1(d7))	640.6	369.3	80	26
CE(18:1(d7))	N/A	675.2	369.4	80	26
CE(18:1)	CE(18:1(d7))	668.6	369.3	80	26

CE(18:2)	CE(18:1(d7))	666.6	369.3	80	26
CE(20:4)	CE(18:1(d7))	690.6	369.3	80	26
CE(20:5)	CE(18:1(d7))	688.6	369.3	80	26
CE(22:6)	CE(18:1(d7))	714.6	369.3	80	26
Cer(d18:0/16:0)	Cer(d18:1/16:0(d7))	540.6	284.3	80	40
Cer(d18:0/18:0)	Cer(d18:1/16:0(d7))	568.7	284.3	80	40
Cer(d18:0/24:0)	Cer(d18:1/16:0(d7))	652.9	284.3	80	40
Cer(d18:0/24:1)	Cer(d18:1/16:0(d7))	650.9	284.4	80	40
Cer(d18:1/16:0(d7))	N/A	545.5	271.4	80	40
Cer(d18:1/16:0)	Cer(d18:1/16:0(d7))	538.5	264.3	80	40
Cer(d18:1/18:0)	Cer(d18:1/16:0(d7))	566.6	264.3	80	40
Cer(d18:1/24:0)	Cer(d18:1/16:0(d7))	650.6	264.3	80	40
Cer(d18:1/24:1)	Cer(d18:1/16:0(d7))	648.6	264.3	80	40
Cholesterol	Cholesterol(d7)	369.3	369.3	80	10
Cholesterol(d7)	N/A	376.2	376.2	80	10
Cholesteryl glucoside	CE(18:1(d7))	566.6	369.3	80	17
Coenzyme Q10	TG(15:0/18:1(d7)/15:0)	863.3	197.2	100	35
DG(15:0/18:1(d7))	N/A	605.6	346.5	80	30
DG(16:0_18:1)	DG(15:0/18:1(d7))	612.4	313.3	80	30
DG(16:0_20:4)	DG(15:0/18:1(d7))	634.5	313.3	80	30
DG(18:0_18:1)	DG(15:0/18:1(d7))	640.4	341.3	80	30
DG(18:0_20:4)	DG(15:0/18:1(d7))	662.5	341.3	80	30

DG(18:0_22:6)	DG(15:0/18:1(d7))	686.6	341.3	80	30
DG(18:1_20:4)	DG(15:0/18:1(d7))	660.5	339.3	80	30
DG(18:1/18:1)	DG(15:0/18:1(d7))	638.4	339.3	80	30
GB3(d18:1/16:0)	GB3(d18:1/18:0(d3))	1025	520.5	80	40
GB3(d18:1/18:0(d3))	N/A	1056	551.6	80	40
GB3(d18:1/18:0)	GB3(d18:1/18:0(d3))	1053	548.6	80	40
GB3(d18:1/24:0)	GB3(d18:1/18:0(d3))	1137	632.6	80	40
GB3(d18:1/24:1)	GB3(d18:1/18:0(d3))	1135	630.6	80	40
GlcCer(d18:1(d5)/18:0)	N/A	733.6	269.3	80	45
GlcCer(d18:1/12:0)	N/A	644.5	264.3	80	40
GlcCer(d18:1/16:0(d3))	N/A	703.7	264.3	80	51
Glucosylsphingosine(d5)	N/A	467.2	269.3	45	16
HexCer(d18:1/16:0)	GlcCer(d18:1(d5)/18:0)	700.6	264.3	80	40
HexCer(d18:1/18:0)	GlcCer(d18:1(d5)/18:0)	728.6	264.3	80	40
HexCer(d18:1/22:0)	GlcCer(d18:1(d5)/18:0)	784.7	264.4	80	40
HexCer(d18:1/24:0)	GlcCer(d18:1(d5)/18:0)	812.7	264.3	80	40
HexCer(d18:1/24:1)	GlcCer(d18:1(d5)/18:0)	810.7	264.3	80	40
Hexosylsphingosine	Glucosylsphingosine(d5)	462.3	264.2	45	16
LacCer(d18:1/16:0)	LacCer(d18:1/17:0)	862.6	264.3	80	40
LacCer(d18:1/17:0)	N/A	876.6	264.3	80	40
LacCer(d18:1/18:0)	LacCer(d18:1/17:0)	890.7	264.3	80	40
LacCer(d18:1/24:0)	LacCer(d18:1/17:0)	974.8	264.3	80	40

LacCer(d18:1/24:1)	LacCer(d18:1/17:0)	972.7	264.3	80	40
Lactosylsphingosine	Glucosylsphingosine(d5)	624.4	264.3	45	16
LPC(16:0)	LPC(18:1(d7))	496.3	184.1	80	40
LPC(16:1)	LPC(18:1(d7))	494.5	184.1	80	40
LPC(18:0)	LPC(18:1(d7))	524.3	184.1	80	40
LPC(18:1(d7))	N/A	529.3	184.1	80	40
LPC(18:1)	LPC(18:1(d7))	522.3	184.1	80	40
LPC(20:4)	LPC(18:1(d7))	544.3	184.1	80	40
LPC(22:6)	LPC(18:1(d7))	568.3	184.1	80	40
LPC(24:0)	LPC(18:1(d7))	608.5	184.1	80	40
LPC(24:1)	LPC(18:1(d7))	606.5	184.1	80	40
LPC(26:0)	LPC(18:1(d7))	636.5	104.1	80	40
LPC(26:1)	LPC(18:1(d7))	634.5	104.1	80	40
lyso-GB3-d7	N/A	793.5	271.3	50	46
lyso-GB3	lyso-GB3-d7	786.6	264.3	50	46
lyso-GB4	GB3(d18:1/18:0(d3))	990.6	264.3	50	52
MG(16:0)	MG(18:1(d7))	348.3	239.3	80	22
MG(16:1)	MG(18:1(d7))	346.3	237.3	80	22
MG(18:0)	MG(18:1(d7))	376.3	267.3	80	22
MG(18:1(d7))	N/A	381.3	272.5	80	22
MG(18:1)	MG(18:1(d7))	374.3	265.3	80	22
MG(20:4)	MG(18:1(d7))	396.3	287.3	80	22

N-Oleoylethanolamine	LPC(18:1(d7))	326.3	62.1	80	23
N-Palmitoyl-O-phosphocholineserine	LPC(18:1(d7))	509.5	184.1	80	40
Palmitoylethanolamine	LPC(18:1(d7))	300.3	62.1	80	23
PC(15:0/18:1(d7))	N/A	754.6	184.1	80	40
PC(16:0/5:0(CHO))	PC(15:0/18:1(d7))	594.5	184.1	80	40
PC(16:0/9:0(CHO))	PC(15:0/18:1(d7))	650.4	184.1	80	40
PC(16:0/9:0(COOH))	PC(15:0/18:1(d7))	666.4	184.1	80	40
PC(18:0/20:4(OH))	PC(15:0/18:1(d7))	826.6	184.1	80	40
PC(18:0/20:4(OOH))	PC(15:0/18:1(d7))	842.6	184.1	80	40
PC(34:1)	PC(15:0/18:1(d7))	760.6	184.1	80	40
PC(36:1)	PC(15:0/18:1(d7))	788.6	184.1	80	40
PC(36:2)	PC(15:0/18:1(d7))	786.6	184.1	80	40
PC(36:4)	PC(15:0/18:1(d7))	782.6	184.1	80	40
PC(38:4)	P C(15:0/18:1(d7))	810.6	184.1	80	40
PC(38:6)	PC(15:0/18:1(d7))	806.6	184.1	80	40
PC(40:5)	PC(15:0/18:1(d7))	836.6	184.1	80	40
PC(40:6)	PC(15:0/18:1(d7))	834.6	184.1	80	40
PC(O-16:0/0:0)	LPC(18:1(d7))	482.3	184.1	80	40
PC(O-16:0/2:0)	LPC(18:1(d7))	524.3	184.2	80	40
PC(O-18:0/2:0)	LPC(18:1(d7))	552.5	184.1	80	40
PE(15:0/18:1(d7))	N/A	711.6	570.5	80	40
PE(18:0/20:4(OH))	PE(15:0/18:1(d7))	784.5	643.4	80	40

PE(18:0/20:4(OOH))	PE(15:0/18:1(d7))	800.5	659.4	80	40
PE(34:1)	PE(15:0/18:1(d7))	718.6	577.6	80	40
PE(36:1)	PE(15:0/18:1(d7))	746.6	605.5	80	40
PE(36:2)	PE(15:0/18:1(d7))	744.6	603.5	80	40
PE(36:4)	PE(15:0/18:1(d7))	740.6	599.5	80	40
PE(38:4)	PE(15:0/18:1(d7))	768.6	627.5	80	40
PE(38:5)	PE(15:0/18:1(d7))	766.6	625.5	80	40
PE(38:6)	PE(15:0/18:1(d7))	764.6	623.5	80	40
PE(40:4)	PE(15:0/18:1(d7))	796.6	655.5	80	40
PE(40:5)	PE(15:0/18:1(d7))	794.6	635.5	80	40
PE(40:6)	PE(15:0/18:1(d7))	792.6	651.5	80	40
PE(40:7)	PE(15:0/18:1(d7))	790.6	649.5	80	40
Sitosteryl glucoside	CE(18:1(d7))	594.6	397.4	80	17
SM(d18:1(d9)/18:1)	N/A	738.7	184.1	80	40
SM(d18:1/16:0)	SM(d18:1(d9)/18:1)	703.6	184.1	80	40
SM(d18:1/18:0)	SM(d18:1(d9)/18:1)	731.6	184.1	80	40
SM(d18:1/24:0)	SM(d18:1(d9)/18:1)	815.7	184.1	80	40
SM(d18:1/24:1)	SM(d18:1(d9)/18:1)	813.7	184.1	80	40
Sphinganine	Sphingosine(d17:1)	302.2	284.3	80	20
Sphinganine 1-phosphate	Sphingosine 1-phosphate-d7	382.3	284.3	80	18
Sphingosine	Sphingosine(d17:1)	300.2	264.3	80	20
Sphingosine 1-phosphate	Sphingosine 1-phosphate-d7	380.3	264.3	80	25

Sphingosine 1-phosphate-d7	N/A	387.3	271.3	80	25
Sphingosine 1-phosphocholine	LPC(18:1(d7))	465.5	184.1	80	40
Sphingosine(d17:1)	N/A	286.2	250.3	80	20
TG(15:0/18:1(d7)/15:0)	N/A	829.8	570.8	80	40
TG(18:0_36:2)	TG(15:0/18:1(d7)/15:0)	904.7	603.4	80	40
TG(18:1_34:2)	TG(15:0/18:1(d7)/15:0)	874.7	575.4	80	40
TG(18:1_34:3)	TG(15:0/18:1(d7)/15:0)	872.7	573.4	80	40
TG(20:4_32:1)	TG(15:0/18:1(d7)/15:0)	870.6	549.3	80	40
TG(20:4_34:2)	TG(15:0/18:1(d7)/15:0)	896.6	575.3	80	40
TG(20:4_34:3)	TG(15:0/18:1(d7)/15:0)	894.6	573.3	80	40
TG(20:4_36:0)	TG(15:0/18:1(d7)/15:0)	928.8	607.5	80	40
TG(20:4_36:2)	TG(15:0/18:1(d7)/15:0)	924.7	603.4	80	40
TG(20:4_36:3)	TG(15:0/18:1(d7)/15:0)	922.7	601.4	80	40
TG(22:6_36:2)	TG(15:0/18:1(d7)/15:0)	948.7	603.4	80	40
TG(22:6_38:1)	TG(15:0/18:1(d7)/15:0)	978.7	633.4	80	40
TG(22:6_38:2)	TG(15:0/18:1(d7)/15:0)	976.7	631.4	80	40

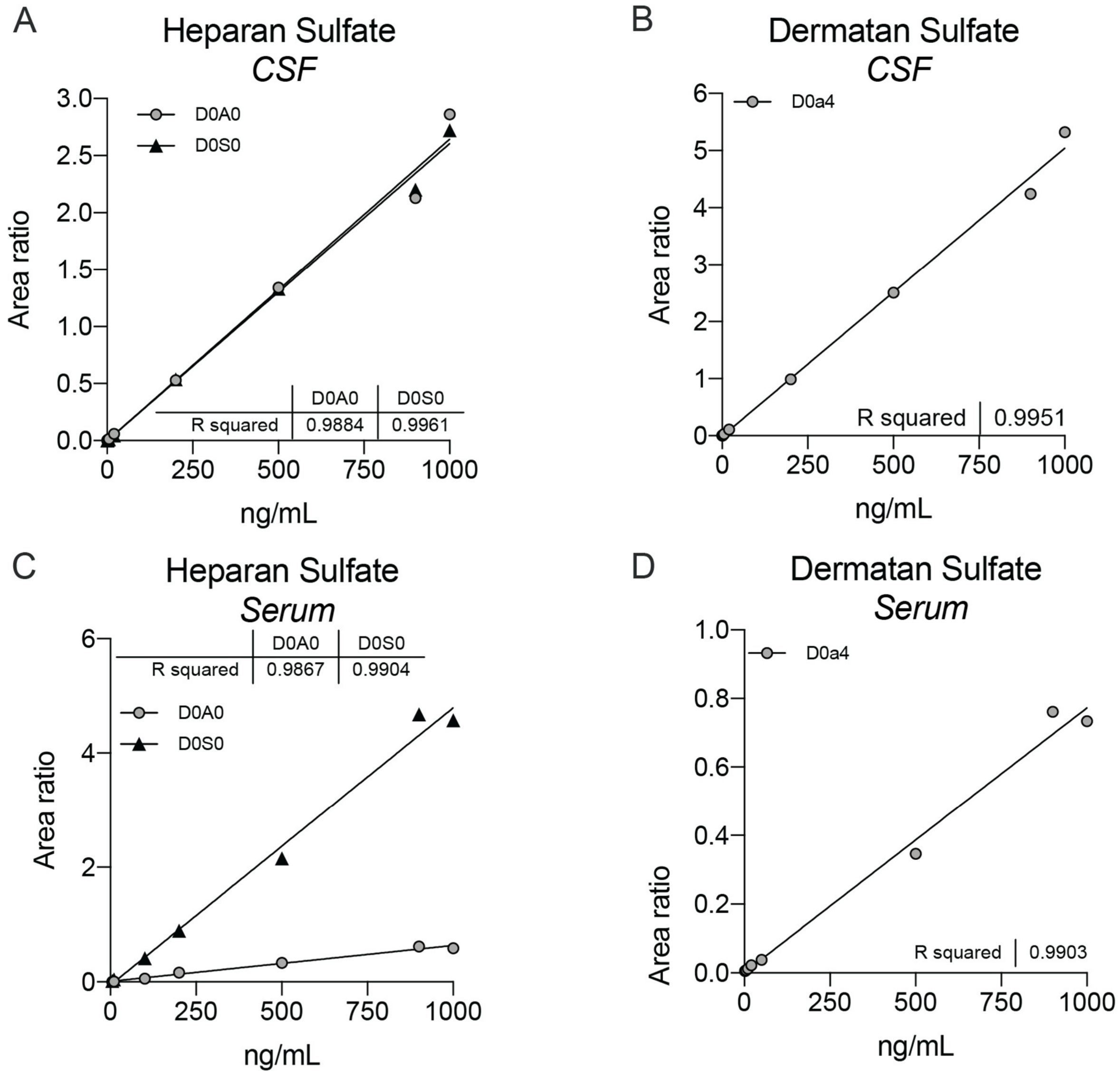

Figure S1. Standard curves for HS and DS measurements in human CSF and serum. CSF and Serum Heparan and dermatan sulfate levels were measured by LC-MS/MS and quantified against calibration curves generated using pure reference standards for D0A0, D0S0, and D0a4. Std curves were generated by spiking standards into CSF and serum matrix in the absence of digesting enzymes followed by LC-MS/MS detection. R squared depicts the fit using a simple linear regression model.

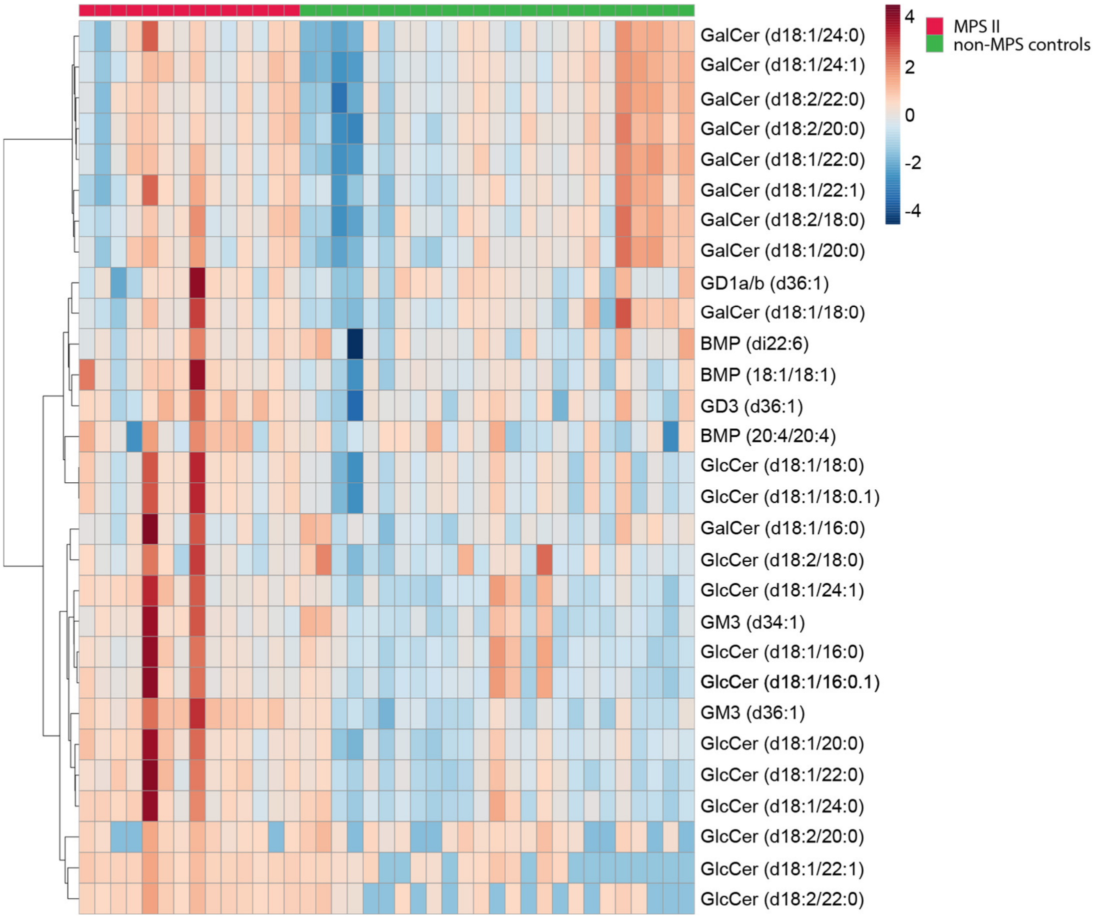

Figure S2. Heatmap of lysosomal lipids. Heatmap was generated for lysosomal lipids between groups. Area ratio for each analyte was used to generate the heatmap. Distance measured using Euclidean, and clustering algorithm using Ward's linkage.

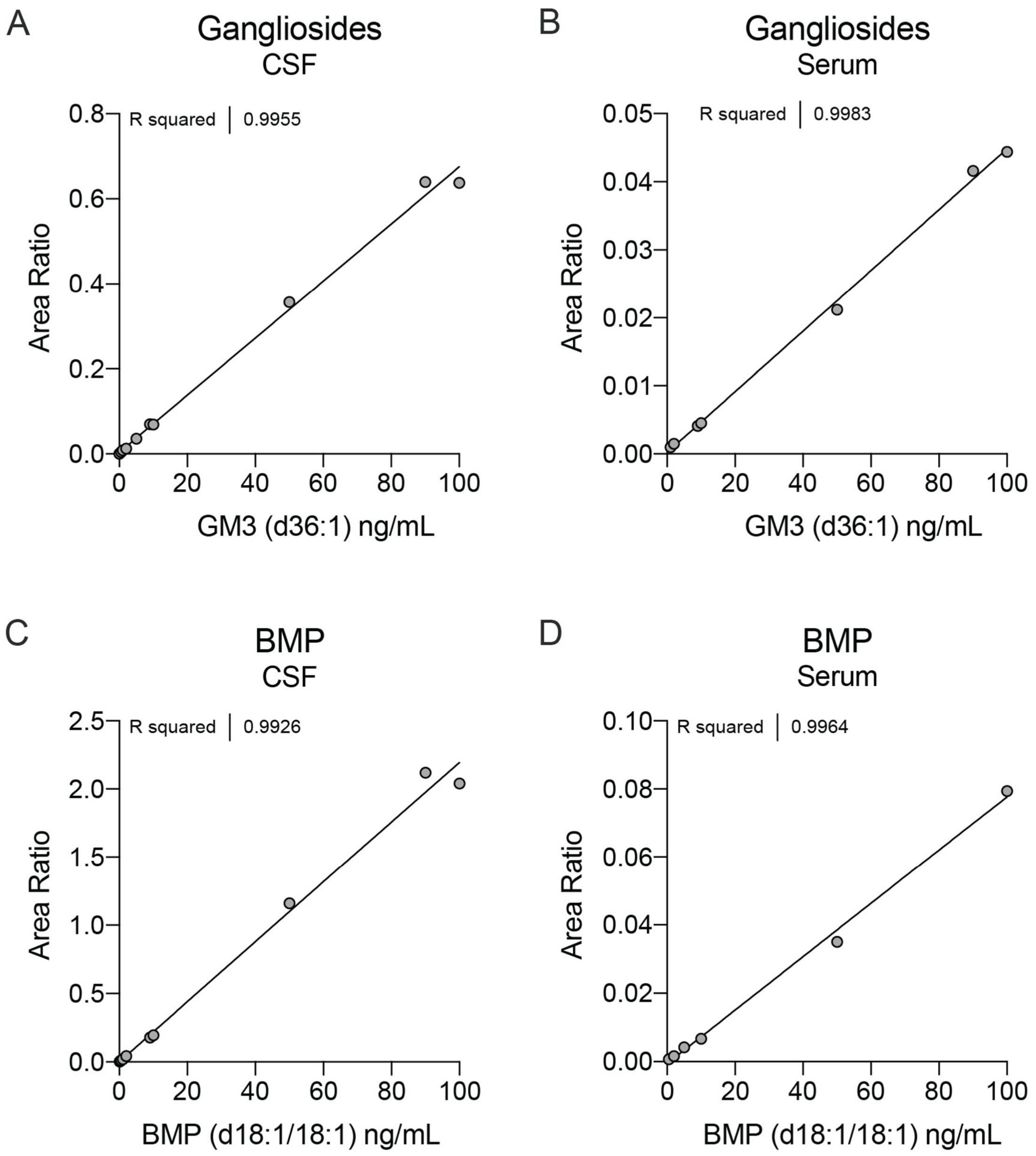

Figure S3. Standard curves for BMP and Gangliosides in human CSF and serum. Quantitative assays for specific lysosomal lipids was developed using mass spec. Calibration curves were generated using pure reference standards for BMP (d18:1/18:1) and GM3 (d36:1). Curves were generated by spiking standards into CSF and serum matrix followed by LC-MS/MS detection to account for matrix effects. R squared depicts the fit using a simple linear regression model.
